# Conservative endometrioma surgery: The combined technique versus CO_2_-laser vaporization only (BLAST: Belgium LAser STudy): Clinical protocol for a multicenter randomized controlled trial

**DOI:** 10.1371/journal.pone.0315709

**Published:** 2025-03-06

**Authors:** Celine Bafort, Sharon Lie Fong, Steffen Fieuws, Brecht Geysenbergh, Michelle Nisolle, Jean-Luc Squifflet, Linda Tebache, Christine Wyns, Christel Meuleman, Carla Tomassetti

**Affiliations:** 1 Department of Obstetrics and Gynecology, Leuven University Fertility Center, University Hospitals Leuven, Leuven, Belgium; 2 Department of Development and Regeneration, KU Leuven, Leuven, Belgium; 3 Department of Public Health, Interuniversity Centre for Biostatistics and Statistical Bioinformatics, KU Leuven, Leuven, Belgium; 4 Department of Obstetrics and Gynecology, GZA (Gasthuiszusters Antwerpen) Sint-Augustinus, Antwerp, Belgium; 5 Department of Obstetrics and Gynecology, Hôpital de La Citadelle, Liège, Belgium; 6 Department of Obstetrics and Gynecology, Cliniques Universitaires Saint-Luc, Brussels, Belgium; 7 Department of Obstetrics and Gynecology, Clinique André Renard, Herstal, Belgium; Dipartimento di Scienze Mediche e Chirugiche (DIMEC), Orsola Hospital, ITALY

## Abstract

**Background:**

The surgical management of endometrioma(s) remains challenging. Although laparoscopic surgery is a well-established treatment of endometrioma(s), caution is required to minimize ovarian damage. Several surgical techniques have been described to treat endometrioma(s): classical cystectomy, ablative techniques, or a combination of both. As cystectomy is strongly associated with a reduction in ovarian reserve, this randomized controlled trial (RCT) aims to determine to what extent the two other surgical procedures may affect ovarian reserve by comparing changes in serum anti-Müllerian hormone (AMH) levels concentrations after each type of surgery.

**Methods:**

This is a multicenter, non-blinded, RCT with parallel groups (group 1 (combined technique) versus group 2 (CO2 laser vaporization only)) and allocation 1:1. Four Belgian centers will be involved. Main inclusion criteria are symptomatic patients (pain and/or infertility), 18–40 years (both inclusive) with an endometriotic cyst (mean diameter of ≥ 2.5 cm and ≤ 8 cm) and AMH level ≥ 0.7 ng/mL. Suspicion of malignancy, a contralateral endometrioma of > 2 cm, use of gonadotrophin-releasing hormone (GnRH) analogues around timing of surgery or previous oophorectomy are exclusion criteria. The primary aim is the evaluation of the difference in serum AMH levels between baseline and 3 months postoperatively (or delta AMH). The secondary outcomes include differences in AMH levels at 6 and 12 months postoperatively, cyst recurrence rate, evolution of pain pattern and fertility outcomes.

**Discussion:**

The present study will help us to answer the question on which surgical technique for endometrioma(s) has the most favorable outcome in patients wishing to preserve their reproductive potential.

**Trial registration:**

ClinicalTrials.gov: NCT04151433. Registered on November 5^th^, 2019.

## Introduction

Endometriosis is defined by the presence of endometrium-like epithelium and/or stroma outside the endometrium and myometrium, usually with an associated inflammatory process [1]. This chronic disease affects 2 to 10% of women of reproductive age and its prevalence increases up to 50% in women with infertility [2,3]. Endometrioma(s) are present in 17 to 44% of patients with the disease [4,5]. Laparoscopic surgery is a well-established treatment of endometrioma(s) however caution is required to minimize ovarian damage [3].

Surgical treatment of endometrioma(s) can be performed by several techniques [1]: **cystectomy**, i.e., excision of the cyst wall; **ablation** using CO_2_-laser vaporization or plasma energy to destroy the inner surface of the cyst wall in situ; or a **partial ovarian cystectomy** combining excisional and ablative surgery. The latter technique will hereafter be referred to as “the combined technique”, which includes first of stripping 80–90% of the cyst wall surface, followed by a second step consisting of ablation of the remaining 10–20% cyst surface attached to the ovarian vascular hilus [1,6]. Although the technique of **coagulation or fulguration** (with destruction of the inner surface of the cyst wall using electrosurgery) has been described before, we will not further discuss this technique since the last ESHRE guideline strongly recommend cystectomy above coagulation or fulguration in terms of recurrence and endometriosis-associated pain [3].

Cystectomy has been the first line surgical treatment for a long time [7,8]. However, based on findings of studies on detrimental effect on ovarian reserve due to endometrioma surgery, alternative techniques should be envisaged, as mentioned in the last ESHRE guideline where CO_2_-laser vaporization is suggested as an alternative to cystectomy [3]. More specifically, a small randomized controlled trial (RCT) by Tsolakidis et al. [9] showed that endometrioma surgery by CO2-laser vaporization (as part of three stage management [10]) had a lower impact on ovarian reserve (measured by decline in AMH) than cystectomy. The RCT by Candiani et al [11] directly compared cystectomy with ‘one-step’ CO_2_-laser ablation and showed a higher AFC after CO_2_-laser ablation. In this same RCT, better results were seen for AMH levels postoperatively in the CO_2_-laser ablation group (no reduction versus significant reduction in the cystectomy group). The combined technique has also been proposed as an alternative technique for the classical cystectomy. In comparison with the contralateral normal ovary, no difference in ovarian volume and AFC was seen postoperatively suggesting that this technique has limited deleterious effect on the ovarian reserve [6].

The RCTs of Tsolakidis and Candiani have used serum anti-Müllerian hormone levels to describe the ovarian reserve. Indeed, serum AMH is the most accurate marker of ovarian reserve [12]. In addition, a recent systematic review and meta-analysis suggested that in women with endometrioma, AMH levels may be of greater utility than AFCs in the assessment of the risk of iatrogenic depletion of the ovarian reserve. This was based on the observation of a significant reduction in AMH levels (which were consistent at the early- intermediate- and late- postoperative time points) after cystectomy but not for AFC (8).

Since CO_2_-laser vaporization and the combined technique may be safer for the normal ovarian tissue as opposed to cystectomy, they are considered as more conservative surgical techniques. In patients wishing to preserve their reproductive potential, the least harmful technique should be preferred when planning ovarian surgery. However, to the best of our knowledge, these different conservative techniques have not been compared directly regarding their effect on ovarian reserve and recurrence rate. We therefore designed this multicenter, non-blinded, RCT with parallel groups and allocation 1:1.

We aimed to determine whether and to what extent these two surgical procedures for endometrioma(s) (combined technique (group 1) versus CO_2_ laser vaporization (group 2)) may affect ovarian reserve by comparing changes in serum AMH levels concentrations after treatment.

## Materials and methods

This study protocol used the SPIRIT 2013 checklist (see S1 File): recommended items to address in a clinical trial protocol and related documents.

### Setting

This is a multicenter national, non-blinded, randomized controlled trial with parallel groups and allocation 1:1. In group 1 a combined technique will be performed versus a complete CO2 laser vaporization in group 2. Four different centers in Belgium will be involved of which the first three are university hospitals:

1. University Hospitals Leuven (Leuven, Belgium)
2. Hôpital de La Citadelle (Liège, Belgium)
3. Cliniques Universitaires Saint-Luc (Brussels, Belgium)
4. GZA (Gasthuiszusters Antwerpen) Sint-Augustinus (Antwerp, Belgium)

Patients are randomly assigned according to a computer-generated randomization list using the method of block randomization (using varying block sizes) to allocate them in a 1:1 ratio. Randomization will be done maximally 2 months before the intervention and minimally 1 hour before start of the intervention by the (sub)investigator of each center. Block randomization per study center will be used to ensure allocation of equal numbers of subjects in each group per center.

### Participants

Patients planned for laparoscopic surgery for endometriotic cysts are eligible to participate in the study. Diagnosis of the endometrioma(s) will be done using transvaginal ultrasound by an experienced sonographer following the International Ovarian Tumor Analysis (IOTA) criteria for reliable diagnosis of endometriomas in premenopausal women [13]: ground glass echogenicity of the cyst fluid, one to four locules, no papillary projections with detectable blood flow. Further mapping of the endometriosis lesions will be done by transvaginal ultrasound using the International Deep Endometriosis Analysis (IDEA) terminology [14] and complemented by magnetic resonance imaging (MRI) when deemed necessary. Before performing surgery for an endometriotic cyst, AMH level is routinely measured (with Roche ECLIA AMH kit since this assay is available in all participating centers). In women with endometrioma(s), the preoperative measurement of AMH level is good clinical practice to assess the potential risk for iatrogenic premature ovarian insufficiency after surgery [15]. Eligible patients will be informed on the study by their endometriosis surgeon, they will receive a patient information leaflet (providing a plain language text in Dutch, French or English). If they are willing to participate, a written informed consent is signed before enrollment.

To be eligible to participate in this study, a subject must meet all the following criteria:

- Age 18 – 40 years (both inclusive)
- Unilateral endometriotic cysts with a mean diameter of ≥ 2.5 cm and ≤ 8 cm, measured in 3 dimensions.
- Complaining of infertility and/or pain
- BMI ≤ 35 kg/m^2^
- Use of contraception (combined hormonal contraceptives or progestogens), at least 4 weeks prior to surgery
- AMH level ≥ 0.7 ng/mL preoperatively (A circulating AMH level of 0.7 ng/ml has been claimed to be the threshold value for poor ovarian responsiveness to controlled ovarian stimulation [16])

A potential subject who meets any of the following criteria will be excluded from participation in this study:

- Patient preference for incomplete surgery for the pelvis (for example patient request to only treat the endometrioma without the other associated endometriotic lesions if present)
- Contra-indication for the use of contraception (combined hormonal contraceptives or progestogens)
- Use of Gonadotrophin-releasing hormone (GnRH) analogues preoperatively and in the first 3 months postoperatively
- (History of) hysterectomy
- Prior unilateral oophorectomy
- Pituitary/hypothalamic disorders
- Suspected malignancy
- Contralateral endometrioma of > 2 cm
- Pregnancy

Prior ovarian surgery (for endometriosis or other cysts) is not an exclusion criterium (as opposed to oophorectomy) but should be reported.

### Interventions

Patients accepting to enter the study will be randomized between 2 different laparoscopic techniques (both arms are existing and accepted surgical strategies for the treatment of endometriomas):

#### Group 1: The combined technique.

First step consisting of stripping the cyst wall for 80% of the surface, followed by a second step consisting of ablation of the remaining 20% cyst surface attached to the ovarian vascular hilus and left on site [6].

#### Group 2: CO_2_-laser vaporization only.

CO_2_-laser vaporization of the complete inner cystic wall after drainage of the cyst content, irrigation, and inspection of its inner wall. A biopsy of the cyst wall will be sent for routine histologic examination to confirm the diagnosis of endometriosis. Ablation of the entire inner surface of the cyst wall using the CO_2_-laser (Lumenis). Power settings of 30–55 watt for the CO_2_-laser beam and 6–10 watt for CO_2_-fibre (based on animal data) are usually used. The CO_2_-laser will be applied in ‘Surgitouch mode’ so that it can ablate the cyst surface while preserving the underlying healthy tissue [17].

If a small contralateral endometrioma is present (≤ 2 cm) this will be treated by CO_2_-laser vaporization only (independent of randomization). Simultaneous treatment of all visual endometriosis lesions (standard procedure). Operative techniques will be recorded as recommended by the CORDES statement [18].

Cross over is allowed from group 1 (combined technique) to group 2 (CO_2_-laser vaporization) if stripping of 80% of the cyst wall is not possible.

Hormonal contraceptives (combined hormonal contraceptives or progestogens) will be used by the participants for at least 4 weeks preoperatively to avoid the presence of a corpus luteum during surgery. The total duration of use of hormonal treatment will be registered. If there was no desire to conceive postoperatively, advise was given to continue the oral contraceptives postoperatively to reduce risk of recurrence [19].

Blinding of the surgeon is not possible. Blinding of surgeons/patients during postoperative follow-up is not feasible and is assumed not to influence the primary outcome. Indeed, bias due to lack of blinding is expected to be negligible since the primary outcome measured is clear and unambiguous (measurement of AMH level).

Due to divergent timing between screening consultation and surgery, AMH level measurement will be repeated the day before or on the day of surgery (baseline AMH level). After surgery this will be repeated at 3, 6 and 12 months postoperatively. Variables influencing AMH levels will also be registered (age, smoking, use of hormonal treatment and duration, previous ovarian surgery, BMI).

**Fig 1:**
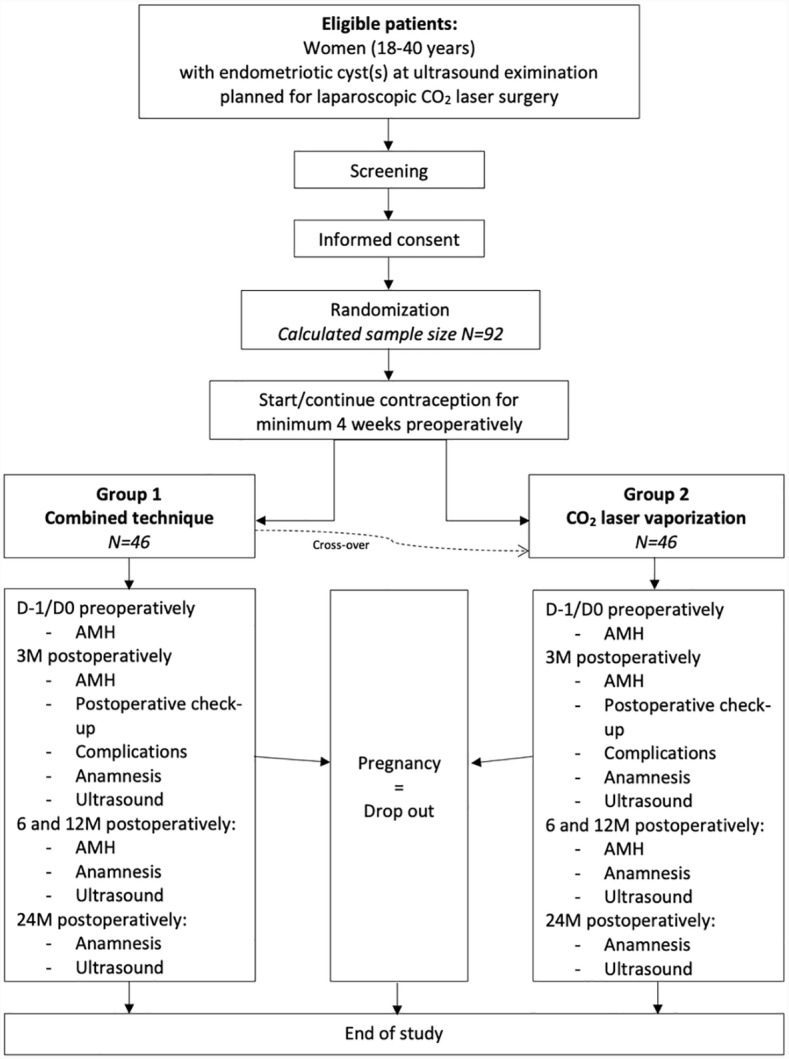
Study flow chart.

**Fig 2:**
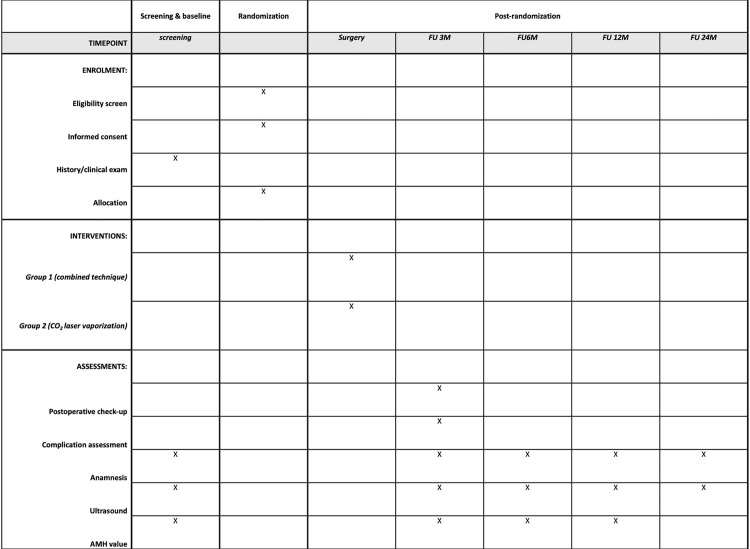
SPIRIT Schedule of enrolment, interventions, and assessments.

Surgical data will be registered including operative time, recording of the hemostatic method used to manage bleeding on an ovary (if necessary), revised American Society for Reproductive Medicine (rASRM) points and stage, Endometriosis Fertility Index (EFI), hospital stay and complications. During postoperative follow-up pain symptoms will be evaluated, transvaginal ultrasound will be performed and AMH levels will be measured at fixed timepoints (3, 6, 12 and 24 months postoperatively, see Figs 1 and 2). In case that a child wish is present, patients will be managed according to their EFI either by non-assisted reproductive technologies (ART) or ART. If a clinical pregnancy occurs postoperatively, the study related follow-up ends but pregnancy outcomes will be recorded.

Both arms of the study are established surgical strategies. Any adverse events will be communicated to the sponsor and principal investigator without undue delay. The adverse events that may occur are those related to surgery and will not differ from what is expected in normal clinical practice. Despite this, a data safety monitoring board (DSMB) has been established including an independent statistician of the KU Leuven to oversee the safety of the participants in the trial.

Patients can leave the study at any time for any reason if they wish to do so without any consequences. The investigator can decide to withdraw a subject from the study for urgent medical reasons. Each withdrawal must be clearly documented. End of study is reached if a patient became pregnant (with referral to an obstetrician for follow-up of the pregnancy) or after completion of the 24 months follow-up period. For follow-up after ending the study, patients were referred to their general gynecologist (not necessarily in the endometriosis unit).

### Study objectives

#### Primary objective.

To assess the effect of conservative surgery of endometrioma(s) on ovarian reserve as reflected by AMH level. For the primary outcome evaluation of serum AMH level measurement will be done before (baseline) and after (at 3 months follow up) laparoscopic treatment of endometrioma(s) (i.e., delta AMH).

The 3 months-period rather than an immediate assessment after surgery was chosen as ovarian surgery inflicts traumatic damage to the ovarian cortex as reflected by a sharp decline in AMH level immediately after surgery, however recovery of the AMH level is expected 3 months postoperatively when edema and local inflammation end [20,21]. The timing of the primary analysis was based on these findings and is similar to the RCT of Candiani [11].

#### Secondary objectives.

AMH difference (or delta AMH)/cyst surface between baseline and at 3-, 6- and 12-months post-operatively with a correction for the cyst surface (since the volume of the cyst may be responsible for more/less influence on the ovarian reserve). The cyst surface will be calculated using the formula for the surface area of a sphere (4 πr^2^). The radius of a sphere is half its diameter, for diameter the mean of the three cysts diameters will be used.

- AMH level modifications (or delta AMH) at 6 and 12 months follow up.
- Cyst recurrence rate at 3, 6, 12 and 24 months postoperatively (visualized by transvaginal ultrasound, any recurrence will be registered).
- Clinical pregnancy (as defined by the International Committee Monitoring Assisted Reproductive Technologies (ICMART) [22,23] as a pregnancy diagnosed by ultrasonographic visualization of one or more gestational sacs or definitive clinical signs of pregnancy. It includes ectopic pregnancy. Note: Multiple gestational sacs are counted as one clinical pregnancy).
- Ectopic pregnancy (as defined by the ICMART [22,23] as a pregnancy in which implantation takes place outside the uterine cavity).
- Miscarriage (defined as a spontaneous loss of pregnancy).
- Live birth (as defined by the ICMART [22,23] as the complete expulsion or extraction from its mother of a product of fertilization, irrespective of the duration of the pregnancy, which, after such separation, breathes or shows any other evidence of life, such as heartbeat, umbilical cord pulsation, or definite movement of voluntary muscles, irrespective of whether the umbilical cord has been cut or the placenta is attached.
- Evolution of pain patterns pre- and postoperatively: each endometriosis related pain complaint will be evaluated using the numerical rating scale (NRS) scale at each visit.
- Premature ovarian insufficiency (POI) postoperatively.

### Data collection and analysis

This study will use an electronic data capture system, i.e., Redcap. Redcap is a web-based system, all study sites will have access to Redcap. The server is hosted within the University Hospitals Leuven and meets hospital level security and back-up. Site access will be controlled, login in Redcap is password controlled. Each user will receive a personal login name and password and will have a specific role which had predefined restrictions on what is allowed in Redcap. Users will only be able to see data of patients of their own site. Any activity in this software is traced and transparent per audit trail and log files. As the randomization will be done in Redcap, a unique study number will be assigned to all subjects and subsequently used in the database. The subject identification code will be safeguarded by the site. The name and any other identifying data will not be included in the study database.

An interim analysis is not planned since both arms are established surgical strategies. The recruitment phase is planned to be 60 months (initially estimated inclusion time of 24 months, due to the COVID-19 pandemic important delay in recruitment for which extension of inclusion time with another 36 months, a notification was made to the Ethical Committee).

Sample size calculation was based on the primary outcome: evaluation of serum AMH level 3 months after laparoscopic treatment of endometrioma(s). Power calculation was based on the findings of the RCT from Candiani et al. [11] where AMH was a secondary outcome in comparing conventional cystectomy versus CO_2_-laser vaporization. In this paper a postoperative AMH of 1,9 ± 0,9 ng/mL was found after CO_2_-laser vaporization only. In this paper a difference in decline in postoperative AMH of 50% was observed in favor of the vaporization group, although this study was not powered for this outcome. Since we will compare two conservative techniques for endometrioma surgery, a difference of 30% AMH decline after surgery was considered to be clinically relevant (after consultation of all participating centers). Based on Candiani et al. (2018) a mean serum AMH of 1.9 (SD = 0.9) ng/mL is expected with CO_2_-laser vaporization. Assuming a common standard deviation a total sample size of 82 patients is needed (or 41 patients in each group) based on a two-sided independent t-test with alpha equal to 0.05 to have at least 80% power to detect a difference of 30% between both groups. To account for the 10% drop out because of pregnancy within 3 months postoperatively, it is prudent to aim for a total sample size of 92 patients (or 46 patients in each group). Note that this calculation is based on the conservative assumption of no correlation between baseline AMH and AMH after 3 months. In practice, the power is expected to exceed largely 80% since the final analysis will be based on an approach taking into account the baseline value. However, since the study wants to gather information on multiple endpoints, we deemed it not appropriate to lower the sample size.

The full analysis set (FAS) will, according to the intent-to-treat principle, include all randomized patients according to their randomized treatment. The FAS will be used for the evaluation of all efficacy endpoints. The primary analysis will be based on the FAS. In case cross-over occurs, an as-treated analysis will be performed additionally. Patients from the FAS with major protocol deviations will be excluded from the per protocol set (PPS). The PPS will be reviewed and finalized prior to database lock at a Blind Review Meeting.

Summary tables (descriptive statistics and/or frequency tables) will be provided for all variables (baseline, surgical and postoperative follow-up). Continuous variables will be summarized with descriptive statistics (n, mean, standard deviation, range, median, p25 and p75). Frequency counts and proportions of subjects within each category will be provided for the categorical data. For the comparison of the mean AMH levels at different timepoints a constrained Longitudinal Data Analysis (cLDA [24]) will be used such that the presence of missing values (due to dropout or to pregnancy, in the latter case AMH values after pregnancy are put on missing) can be handled. Center will be added as a fixed effect in the cLDA model. The same approach will be used for the (log-transformed) ratio of the AMH level and the cyst surface, and for the longitudinally gathered NRS pain scores. Cyst recurrence rate until 24 months postoperatively will be visualized using Kaplan-Meier estimates and compared using a stratified log-rank test.

Subgroup analysis will be performed on patients without previous history of ovarian surgery (if relevant) and depending on continuation of contraception postoperatively.

The datasets, including anonymized patient-level data generated during the current trial, will be available after study completion from the corresponding author upon reasonable request.

### Dissemination plans

Results from this trial will be shared through publications and presented at international conferences. All participating investigators will be co-authors, according to the number of patients included and intellectual contribution.

### Status and timeline of the study

The study protocol was approved by the ethics committee research UZ/KU Leuven on October 24^th^ 2019 (institutional review board (IRB) number: S62899) and registered on clinicaltrials.gov (NCT04151433) on November 5^th^ 2019. All participating patients will sign a written informed consent, as approved by the ethical committees. Coordinating center: University Hospitals Leuven.

Patient recruitment is ongoing and started after approval of the ethics committee (December 1^st^ 2019). Expected end of inclusion will be no later than June 30^th^, 2025. End of study, with collection of all secondary outcomes, is foreseen by mid 2027 (if necessary, extend with 9 months to allow full follow-up of the pregnancies until delivery). Current protocol version is 2.2 (dated 16-12-2021), see S2 File (PDF version). The English informed consent form can be found in S3 File.

## Discussion

Since CO_2_-laser vaporization and the combined technique may be safer for the normal ovarian tissue as opposed to cystectomy, they are considered as more conservative surgical techniques. In patients wishing to preserve their reproductive potential, the least harmful technique should be preferred when planning ovarian surgery. To the best of our knowledge, these different conservative techniques have not been compared directly yet regarding their effect on ovarian reserve and/or disease recurrence.

This study presents some **challenges:**

First, patients may be reluctant to participate in an RCT due to the random allocation to a specific surgical treatment. We experience that random allocation by a computer system might be difficult to accept for our patients, although by explaining that the two proposed surgical techniques in this RCT are both considered to be less harmful for the ovarian reserve than the classical cystectomy [6,9,11] we expect patients to be willing to participate. Next to ovarian reserve, another question that is often asked by patients is risk of recurrence. Short term follow-up (one year postoperatively) shows a higher recurrence rate in the CO_2_-laser vaporization group [25]. When looking at the available evidence on long term recurrence rate, similar recurrence rates have been described after cystectomy and CO_2_-laser vaporization [25,26] or mixed techniques (including the combined technique) [27].

Second is the concern of non-adherence to the scheduled follow-up up until two years postoperatively. However, the primary outcome is measured at 3 months postoperatively simultaneously with the first routine postoperative check-up (no extra hospital visit will be required). Therefore, we expect that the drop-out rate before this timepoint will be low. If a patient becomes pregnant within 3 months postoperatively, the study related follow-up ends (with drop out for the primary outcome), although pregnancy outcomes will be recorded. This was accounted for when calculating our sample size. Finally, previous studies in our group on long term outcomes showed great willingness of endometriosis patients to adhere to study protocols [28,29].

Third is the challenge of performing a multicenter study. Several meetings with the participating centers were organized during protocol development to standardize preoperative treatment and discuss the study flow.

Next to the challenges, the study may have some **limitations:**

First, AMH level was chosen as the marker for ovarian reserve although it is the most appropriate marker available, it is not perfect. Indeed, AMH level can be difficult to interpret due to following reasons: 1) Different reproductive and lifestyle determinants can influence AMH levels as shown by the study of Dólleman et al [30]. 2) Although AMH levels in serum vary significantly across the menstrual cycle (with a slight increase during follicular phase, particularly for women over 30 years), the age-related decline of AMH seems consistent regardless of the menstrual cycle day of the AMH assessment [31]. Therefore, sample collection can be performed on any day of the menstrual cycle for assessment of ovarian reserve. 3) Unknown effect of the use of GnRH analogue on AMH values [32], which led us to exclude its use around the operating time. On the other hand, AMH level measurement also has advantages: 1) It is an easy measurement (by blood sample with a validated assay); 2) It is an objective measurement compared to AFC, AFC is a subjective measurement with inter-operator and inter technical variability based on ultrasound and thus prone to more variable results. Moreover, adequate measurement of AFC is challenging in the presence of a(n) ovarian cyst(s) and in patients with high BMI.

Second, we decide to include a selected group of patients into this study: unilateral endometrioma(s) with an AMH level at screening > 0.7 ng/ml (a small contralateral endometrioma of < 2 cm was allowed). For this reason, our results may not be entirely generalizable for patients with bilateral (large) endometrioma(s) and possibly more extensive disease [33].

Third, we chose to perform a real-life study including both patients with pain and/or infertility. This heterogenous group of patients will differ in postoperative need of contraception, despite advice to continue hormonal therapy postoperatively for secondary prevention of recurrence [19], desire for a natural pregnancy or advice to start a fertility treatment. All these variables will be registered, and certain subgroup analyses are planned (as described above). Notwithstanding, the randomization process should allocate these equally over both study arms.

Overall, we believe that this RCT will add clinically valuable information not only on ovarian reserve but also on recurrence, evolution of pain patterns postoperatively and fertility outcomes. The ultimate goal of this RCT is to contribute significantly to optimized care with regards to surgical techniques for the treatment of endometrioma(s). Observations could then further be integrated in decision algorithms for treatment of patients with endometriomas with (future) fertility wishes. Whereas our focus lies on ovarian reserve, the high quality of the collected data will also allow meta-analysis for the secondary outcomes.

## Supporting information

S1 File SPIRIT 2013 checklist. Recommended items to address in a clinical trial protocol and related documents.
(DOCX)

S2 File Protocol. Original full protocol (PDF).
(PDF)

S3 File Informed consent form template (in English). (PDF)
