# Supplementary material for: Conservative endometrioma surgery: The combined technique versus CO2-laser vaporization only (BLAST: Belgium LAser STudy): Clinical protocol for a multicenter randomized controlled trial
Source: PLoS One. 2025 Mar 6;20(3):e0315709. doi: 10.1371/journal.pone.0315709 (PMC11884717; doi:10.1371/journal.pone.0315709)
Supplement: S2 File — Original full protocol (PDF). (PDF) [file pone.0315709.s002.pdf]

RESEARCH PROTOCOL

(December 2021)

BLAST:

a randomized controlled trial on conservative endometrioma surgery using the CO_2 laser: the combined technique versus CO_2 laser vaporization only

UZ LEUVEN

CHR Liège CITADELLE CHATEAU ROUGE SAINTE ROSALIE
VOTRE SANTÉ, NOTRE MÉTIER

GZA Ziekenhuizen GasthuisZusters Antwerpen
Sint-Augustinus - Sint-Vincentius - Sint-Jozef

Cliniques universitaires SAINT-LUC UCL BRUXELLES

PROTOCOL TITLE ‘BLAST: a randomized controlled trial on conservative endometrioma surgery using the CO_2 laser: the combined technique versus CO_2 laser vaporization only’

**Protocol ID**	**S62899**
**Short title**<br>**Acronym**	**RCT on conservative endometrioma surgery**<br>**BLAST (Belgian LAser STudy)**
**Version**	**2.2**
**Date**	**16-12-2021**
**Coordinating investigator/project leader**	***Prof Dr C. Tomassetti (UZ Leuven - Leuven)***<br>***Dr C. Bafort (UZ Leuven – Leuven)***
**Principal investigators**<br><br>***Sub investigators***	***Prof Dr C. Tomassetti (UZ Leuven - Leuven)***<br>***Dr C. Bafort (UZ Leuven – Leuven)***<br>***Prof Dr C. Meuleman (UZ Leuven – Leuven)***<br>***Prof Dr M. Nisolle (CHR La Citadelle – Liège)***<br>***Dr L. Tebache (CHR La Citadelle – Liège)***<br>***Dr B. Geysenbergh (GZA Sint Augustinus – Antwerp)***<br>***Prof Dr J-L. Squifflet (UCL – Brussels)***<br>***Prof Dr C. Wyns (UCL – Brussels)***
**Sponsor**	***UZ/KU Leuven (Institution)***
**Laboratory sites**	***Blood samples for AMH measurements (using Roche ECLIA AMH kit): standard of care sample will be done at the laboratory of each participating center – study samples will be sent to the laboratory of UZ Leuven***

PROTOCOL SIGNATURE SHEET

Name	Signature	Date
**Sponsor or legal representative:**<br>***Prof Dr W. Robberecht***<br>***UZ Leuven***<br>***Herestraat 49***<br>***3000 Leuven, Belgium***		
**Coordinating Investigator/Project leader/Principal Investigator:**<br>***Prof Dr C. Tomassetti***<br>***Head of department of Fertility and Endometriosis at University Hospitals Leuven***<br>***Dr C. Bafort***<br>***Trainee in gynecology and obstetrics/PhD student***		

PROTOCOL SIGNATURE SHEET PARTICIPATING CENTERS

Protocol title: 'BLAST: a randomized controlled trial on conservative endometrioma surgery using the CO_2 laser: the combined technique versus CO_2 laser vaporization only'

Protocol ID: S62899

Protocol version/date: Version 2.2 – Date 16-12-2021

Name	Signature	Date
**Sponsor or legal representative:**<br>***Prof Dr W. Robberecht***<br>***UZ Leuven***<br>***Herestraat 49***<br>***3000 Leuven, Belgium***		
**Coordinating Investigator/Project leader/Principal Investigator:**<br>***Prof Dr C. Tomassetti***<br>***Head of department of Fertility and Endometriosis at University Hospitals Leuven***<br>***Dr C. Bafort***<br>***Trainee in gynecology and obstetrics/PhD student***		

Declaration of Sub investigator:

I confirm that I have read the above-mentioned protocol and its attachments. I agree to conduct the described trial in compliance with all stipulations of the protocol, regulations and ICH E6 Guideline for Good Clinical Practice (GCP).

Name	Signature	Date
**Celine Bafort**		
**Christel Meuleman**		
**Michelle Nisolle**		
**Linda Tebache**		
**Brecht Geysenbergh**		
**Jean-Luc Squifflet**		
**Christine Wyns**		

TABLE OF CONTENTS

1. INTRODUCTION AND RATIONALE 9
2. OBJECTIVES 11
3. STUDY DESIGN 12
4. STUDY POPULATION 13
4.1 Population (base) 13
4.2 Inclusion criteria 13
4.3 Exclusion criteria 13
4.4 Sample size 14
4.5 Patient drop out/end of study 14
5. TREATMENT OF SUBJECTS 14
5.1 Investigational treatment 14
5.2 Use of co-intervention 15
6. METHODS 16
6.1 Study parameters/endpoints 16
6.1.1 Main study parameter/endpoint 16
6.1.2 Secondary study parameters/endpoints 16
6.1.3 Other study parameters 17
6.2 Randomization, blinding and treatment allocation 19
Patients fitting the inclusion criteria and consenting to participate are randomly assigned according to a computer-generated randomization list using the method of block randomization (using varying block sizes) to allocate them in proportion, at 1:1, either to Group 1 (combined technique) or Group 2 (CO_2 laser vaporization). 19
6.3 Study procedures 19
6.3.1 Participants flow 19
6.3.2 Time requirements 21
6.3.3 Trial monitoring and patient visits 21
6.3.4 Study costs 22
6.3.5 Budget and funding 23
6.4 Withdrawal of individual subjects 23
7. SAFETY REPORTING 23
7.1 Temporary halt for reasons of subject safety 23
7.2 Definitions AEs, SAEs and SUSARs 23
Adverse events (AEs) 23
7.2.1 23
7.2.2 Serious adverse events (SAEs) 23
7.2.3 Suspected Unexpected Serious Adverse Reaction (SUSAR) 24
7.3 Adverse events that do not require reporting 24
7.4 Recording and reporting of Adverse events 25
7.4.1 Assessment 25
7.4.2 Timeline for reporting 26
7.4.3 Follow-up 26

7.4.4 Pregnancy 27
7.4.5 Death 27
7.4.6 Annual reporting 27
7.4.7 Overview reporting requirements 27
7.5 Data Safety Monitoring Board (DSMB) 28
8. STATISTICAL ANALYSIS 28
8.1 Sample size calculation: 28
8.2 Baseline data: 29
8.3 Primary study parameter(s) 31
8.4 Secondary study parameter(s) 32
8.5 Subgroup analysis 32
8.6 Timing of data analysis 32
9. ETHICAL CONSIDERATIONS 33
9.1 Regulation statement 33
9.2 Recruitment and consent 33
9.3 Benefits and risks assessment, group relatedness 34
9.4 Compensation for injury 34
9.5 Incentives 34
10. ADMINISTRATIVE ASPECTS, MONITORING AND PUBLICATION 34
10.1 Handling and storage of data and documents 34
10.2 Monitoring and Quality Assurance 35
10.3 Public disclosure and publication policy 35
11. REFERENCES 35
12. ANNEX: DATA PROCESSING AGREEMENT (“DPA”) 38
12.1 SCOPE OF THE DATA PROCESSING AGREEMENT 38
12.2 PROCESSING OF PERSONAL DATA 38
12.3 THE DATA PROCESSOR'S OBLIGATIONS 38
12.4 SUBPROCESSORS 41
12.5 CONFIDENTIALITY 41
12.6 TERM AND TERMINATION OF THE DATA PROCESSING AGREEMENT 42

LIST OF ABBREVIATIONS AND RELEVANT DEFINITIONS

AE	Adverse Event
AFC	Antral Follicle Count
AMH	Anti-Müllerian Hormone
ART	Assisted reproductive treatment
ASAP	As Soon As Possible
CV	Curriculum Vitae
DPA	Data Processing Agreement
DSMB	Data Safety Monitoring Board
EC	Ethics Committee
EU	European Union
GCP	Good Clinical Practice
GDPR	General Data Protection Regulation; in Dutch: Algemene Verordening Gegevensbescherming (AVG)
IC	Informed Consent
ICSI	Intracytoplasmic sperm injection
IUI	Intra uterine insemination
IVF	In vitro fertilization
LUFC	Leuven University Fertility Center
OCP	Oral contraceptive pill
PCOS	Polycystic ovary syndrome
POI	Premature ovarian insufficiency
RCT	randomized controlled trial
(S)AE	(Serious) Adverse Event
Sponsor	The sponsor is the party that commissions the organization or performance of the research, for example a pharmaceutical company, academic hospital, scientific organization or investigator. A party that provides funding for a study but does not commission it is not regarded as the sponsor, but referred to as a subsidizing party.
SUSAR	Suspected Unexpected Serious Adverse Reaction

SUMMARY

Rationale: To study which technique - CO2 laser vaporization only or the combined technique - offers better results in treating endometriomas in terms of ovarian reserve preservation. Ovarian reserve will be assessed by consecutive measurements of AMH serum levels before and after surgery.

Objective: To test the hypothesis that CO2 laser vaporization only of the cysts will result in less ovarian damage than when the combined technique is used.

Primary outcome: serum AMH three months after laparoscopic treatment of endometrioma(s).

Secondary outcomes will include amongst others serum AMH at later timepoints, cyst recurrence rate and pregnancy rate.

Study design: Multicenter national randomized controlled trial comparing group 1 (combined technique) versus group 2 (CO2 laser vaporization only). Participation of 4 different centers in Belgium: CHR La Citadelle (Liège), GZA Sint-Augustinus (Antwerp), Cliniques universitaires Saint-Luc – UCL (Brussels) and University Hospitals Leuven (Leuven).

Study population: Women (18-40 years) scheduled for laparoscopic CO_2 laser surgery for endometriotic cysts.

Intervention: different techniques in conservative laparoscopic treatment of endometriomas: in group 1 the combined technique will be performed and in group 2 CO_2 laser vaporization only will be done.

Main study parameters/endpoints: the main study parameter is the difference in postoperative AMH.

Nature and extent of the burden and risks associated with participation, benefit and group relatedness: no extra burden (patients are undergoing surgery regardless of participation in the trial), no expected extra risks, no benefit.

1. INTRODUCTION AND RATIONALE

Endometriosis is still an enigmatic disease affecting 6%-10% of women of reproductive age. It is defined by the presence of endometrium-like tissue outside the uterus such as the pelvic peritoneum, ovaries and rectovaginal septum. Symptoms include pain and/or infertility (1, 2). Phenotypically, three types of endometriosis lesions can be distinguished: superficial (mainly peritoneal) endometriosis, deep endometriosis (nodules) and ovarian endometriotic cysts (endometriomas).

Endometriosis may require surgical treatment to address pelvic pain and infertility. Although the surgical (laparoscopic) management of endometriosis is widely accepted, the ideal surgical techniques for treatment of ovarian endometriotic cysts (endometriomas) are still being debated.

Surgical treatment of endometriomas is mainly performed by 2 types of procedures: cystectomy (excision of the cyst wall) and ablation (destruction of the inner surface of the cyst wall in situ). There are two main risks associated with the surgical treatment of endometriomas: 1. removal or destruction of normal ovarian cortex together with the endometrioma with subsequent reduction of ovarian reserve, and 2. incomplete surgery with subsequent early recurrence of endometriomas (2, 3). With the intent of combining the advantages of excisional surgery (cystectomy) in terms of recurrence rates, and those of non-excisional techniques (ablation) in terms of better preservation of the normal ovarian tissue, another surgical technique has been proposed: the combined technique (first step consisting of stripping the cystwall for 80-90% of the surface, followed by a second step consisting of ablation of the remaining 10-20% cyst surface attached to the ovarian vascular hilus and left on site) (4).

Two RCTs compared the recurrence rates after cystectomy with those after endometrioma ablation using bipolar current and concluded that recurrence rate was higher with ablation (5-7). However, these trials used bipolar current for ablation with higher thermal damage compared to CO2 laser technology. CO2 laser has the ability to deliver energy with little thermal spread and subsequently less ovarian tissue damage due to surgery. Ablation with CO2 laser may be less invasive than conventional cystectomy as shown with a more recent RCT showing with increased preservation of antral follicles in favor of ablation (8). Comparable recurrence rates after cystectomy and CO2 laser vaporization were found in other studies (4, 9). To our knowledge, different conservative techniques have not been

compared directly yet regarding their effect on ovarian reserve and/or recurrence (based on a Medline search on ‘RCT’ AND ‘endometrioma’).

Ovarian reserve is defined as the functional potential of the ovary and reflects the number and quality of follicles remaining in woman’s ovaries at any time. Various tests and markers have been reported to report the numbers of remaining follicles: follicle-stimulating hormone (FSH), estradiol, AMH, inhibin-B and sonographic variables such as antral follicle count (AFC). Most widely used markers are AFC and AMH. Although AMH levels in serum vary significantly across the menstrual cycle (with a slight increase during follicular phase, particularly for women over 30 years); the age-related decline of AMH seems consistent regardless of the menstrual cycle day of the AMH assessment(10). Therefore, sample collection can be performed on any day of the menstrual cycle for assessment of ovarian reserve. Secondly it is an easy and objective measurement. AFC has the advantage of showing the reserve of a single ovary while the AMH value reflects the ovarian reserve for both ovaries. However, in case of a(n) ovarian cyst(s) adequate measurement of AFC is challenging because of the presence of this cyst. Next to this it is a subjective measurement and thus prone to more variable results than AMH.

AMH is an ovarian reserve marker and is increasingly applied in the clinical practice as a prognostic tool for ovarian response ART treatment. A low AMH value means a lower ovarian reserve although it cannot predict the age at menopause (11).
Different reproductive and lifestyle determinants can influence AMH levels as shown by the study of Dólleman et al (12). Oral contraceptive pill (OCP) users, women with an irregular cycle (associated with menopausal transition) and pregnant women have a significantly lower age-specific AMH percentiles (for oral contraceptive users and cycle irregularity 11 percentiles lower; for pregnancy 17 percentiles lower). While women with polycystic ovary syndrome (PCOS) have higher age-specific AMH levels.
There was no clear relationship of duration of oral contraceptive use with age-specific AMH levels. Age at menarche and age at first childbirth did not influence AMH percentiles. Current smoking is associated with 4 percentiles lower age-specific AMH percentiles. Other lifestyle factors such as body mass index, alcohol consumption, physical exercise and socioeconomic status were not associated with age-specific AMH percentiles. The lower AMH levels associated with oral contraceptive use and smoking seem to be reversible. It is crucial to consider the effect of those determinants when interpreting AMH in a clinical setting and subsequent counseling of patients. Of the hormonal treatments, not only oral contraceptives influence AMH values but also depot GnRH agonists have an impact on AMH levels

suggesting that AMH may not be a reliable marker of ovarian reserve under administration of GnRH agonists (13).
Hormonal treatment is often used preoperatively to reduce endometriosis associated pain in the waiting period before the surgery can be performed. In clinical practice, surgeons prescribe preoperative medical treatment with GnRH analogues as this can facilitate surgery due to reduced inflammation, vascularization of endometriosis lesions and adhesions. However, there are no controlled studies supporting this (14). Oral contraceptives are used in the preoperative period for pain relief in the preoperative period and contraceptive function (14).

The paper of Chang et al (15), where changes in ovarian reserve after laparoscopic cystectomy were evaluated pre- and postoperatively, suggest that ovarian reserve could be reduced after laparoscopic cystectomy, however it could be restored after up to 3 months postoperative. AMH, next to being a marker of ovarian reserve, can also be used as a marker for surgical damage to the ovaries.

The aim of this study is to determine whether and to what extent the two surgical procedures for endometriomas treatment (combined technique versus CO_2 laser vaporization) may affect ovarian reserve by comparing changes in serum AMH concentrations after treatment.

2. OBJECTIVES

Primary Objective: To assess the effect of conservative laparoscopic treatment of endometrioma(s) on ovarian reserve as reflected by AMH in patients planned for laparoscopic CO_2 laser surgery. For the primary outcome evaluation of serum AMH will be done before (baseline) and after (at 3 months follow up) laparoscopic treatment of endometrioma(s).

The primary study hypothesis is that CO_2 laser vaporization only of the cyst wall will result in less ovarian damage than when the combined technique is performed. The null hypothesis is that CO_2 laser vaporization of the cyst wall does not result in better preservation of the ovarian reserve.

AMH was chosen as marker of the ovarian reserve since it is the only marker being menstrual cycle independent and easily measured. AMH measurements will be analyzed using the Roche ECLIA AMH kit (available in all centers). Multiple determinants (as

described above) influence AMH values, ideally AMH measurement is done in a natural regular cycle of non-smoking and non-pregnant women. In the daily clinical practice this is not always the case due to the variations in patients' clinical presentations.

Secondary Objectives:

- AMH difference/cyst surface
  between baseline and at 3, 6 and 12 months post-operative with a correction for the cyst surface (since the volume of the cyst may be responsible for more/less influence on the ovarian reserve)
- AMH modifications at 6 and 12 months follow up
- Cyst recurrence rate at 3, 6, 12 and 24 months postoperatively (visualized by transvaginal ultrasound)
- Clinical pregnancy (as defined by the ICMART (16) as a pregnancy diagnosed by ultrasonographic visualization of one or more gestational sacs or definitive clinical signs of pregnancy. It includes ectopic pregnancy. Note: Multiple gestational sacs are counted as one clinical pregnancy.)
- Ectopic pregnancy (as defined by the ICMART (16) as a pregnancy in which implantation takes place outside the uterine cavity)
- Miscarriage (defined as a spontaneous loss of pregnancy)
- Live birth (as defined by the ICMART (16) as the complete expulsion or extraction from its mother of a product of fertilization, irrespective of the duration of the pregnancy, which, after such separation, breathes or shows any other evidence of life, such as heart beat, umbilical cord pulsation, or definite movement of voluntary muscles, irrespective of whether the umbilical cord has been cut or the placenta is attached.
- Evolution of pain patterns pre- and postoperatively: each endometriosis related pain complaint will be evaluated using the NRS scale at each visit.
- POI postoperatively

3. STUDY DESIGN

Multicenter national randomized controlled trial comparing two types of conservative ovarian surgery for endometriotic cysts:

- group 1: the combined technique
- group 2: CO_2 laser vaporization only

Participation of 4 different centers in Belgium:

1. CHR La Citadelle (Liège)

2. GZA Sint-Augustinus (Antwerp)
3. University Hospitals Leuven (Leuven)
4. Cliniques universitaires Saint- Luc - UCL (Brussels)

All participating surgeons master both techniques equally well.

4. STUDY POPULATION

4.1 Population (base)

Patients planned for laparoscopic CO_2 laser surgery for endometriotic cysts. Diagnosis of the endometrioma(s) using transvaginal ultrasound by an experienced sonographer. The patients must fit the International Ovarian Tumor Analysis (IOTA)-criteria for reliable diagnosis of endometriomas in premenopausal women (17): ground glass echogenicity of the cyst fluid, one to four locules, no papillations with detectable blood flow.

4.2 Inclusion criteria

In order to be eligible to participate in this study, a subject must meet all of the following criteria:

- Age: 18 – 40 years (both inclusive)
- Unilateral endometriotic cysts with a mean diameter of ≥2.5cm and ≤8 cm, measured in 3 dimensions.
- Presence of a contralateral endometrioma of ≤ 2cm is allowed
- Complaining of infertility and/or pain
- BMI ≤35
- Use of contraception OCP? (combined or Progesteron only) for at least 4 weeks before surgery

4.3 Exclusion criteria

A potential subject who meets any of the following criteria will be excluded from participation in this study:

- Incomplete surgery for the pelvis
- Contra-indication for the use of contraception (combined or Progesteron only)
- Use of GnRH analogues preoperatively and in the first 3 months postoperatively
- (History of) hysterectomy
- Prior unilateral oophorectomy
- Pituitary/hypothalamic disorders
- Suspected malignancy

- Contralateral endometrioma of ≥2 cm
- AMH <0.7 preoperatively (A circulating AMH level of 0.7 ng/ml has been claimed to be the threshold value for poor ovarian responsiveness to controlled ovarian stimulation (18))
- Pregnancy

Prior ovarian surgery is allowed (for endometriosis or other cysts) but should be reported.

4.4 Sample size

Sample size calculation is based on the primary outcome: evaluation of serum AMH 3 months after laparoscopic treatment of endometrioma(s). Total sample size of 92 patients taken into account 10% drop out because of pregnancy within 3 months postoperatively (cf. page 28 – statistical analysis for detailed information).

4.5 Patient drop out/end of study

If a patient becomes pregnant (clinical pregnancy) within 3 months postoperatively:
- drop-out of the study for primary outcome (data cannot be included for primary outcome studied)
- follow up of the pregnancy outcome

If a patient is pregnant (clinical pregnancy) after 3 months postoperatively:
- data can still be included for the primary outcome studied
- secondary outcomes can be studied until drop out
- follow up of the pregnancy outcome

AMH measurements only outside pregnancy.

5. TREATMENT OF SUBJECTS

5.1 Investigational treatment

Comparison between 2 different laparoscopic techniques in conservative surgical treatment of endometriomas (both arms are existing and accepted surgical strategies):
- Group 1: the combined technique: first step consisting of stripping the cyst wall for 80% of the surface, followed by a second step consisting of ablation of the remaining 20% cyst surface attached to the ovarian vascular hilus and left on site (4)
- Group 2: CO_2 laser vaporization only of the complete inner cystic wall after drainage of the cyst content, irrigation and inspection of its inner wall. A biopsy of the cyst wall was

sent for routine histologic examination to confirm the diagnosis of endometriosis. Ablation of the entire inner surface of the cyst wall using the CO_2 laser (Lumenis). Power settings of 30–55 W for CO2 laser beam and 6–10 W for CO2 fibre (based on animal data) are usually used. The laser should be on the ablate function to widen the beam (e.g. ‘Surgitouch modus’). The laser should be applied in Surgitouch mode so that it can ablate the cyst surface while preserving the underlying healthy tissue. (19)

Intention to treat analysis. Cross over is allowed from group 1 (combined technique) to group 2 (CO_2 laser vaporization) if stripping of 80% of the cyst wall is not possible.

If a small contralateral endometrioma is present (≤ 2cm) this will be treated by CO_2 laser vaporization only (independent of randomization).

Simultaneous treatment of all visual endometriosis lesions (standard procedure). Operative techniques should be recorded as recommended by the CORDES statement (20).

All patients included in the study undergo surgery after administration of hormonal contraceptives (standard of care). After consultation of all participating centers the decision was made of using oral contraceptives during minimally 4 weeks preoperatively. Use of GnRH agonist is an exclusion criterium since we cannot predict the effect on AMH values. If no child wish was present, advise was given to continue the oral contraceptives postoperatively.

5.2 Use of co-intervention

Use of contraception pre-surgery:

- If patients already use some form of hormonal contraception: continuation of the treatment.
- If patients have natural cycle: start hormonal contraception during next menstruation for at least 4 weeks preoperatively

Duration of use of the hormonal treatment will be registered.

Types of allowed contraception:

- Combined oral contraception (classic pil)
- Vaginal ring (eg Nuvaring, Izzyring,…)
- Progesteron only pil

- Hormone releasing intrauterine device (for example: Mirena, Kyleena,…) are allowed provided that they are combined with oral/vaginal contraception for adequate ovariostasis

The use of GnRH analogues (both preoperatively and in the first 3 months postoperatively) is not allowed.

Other chronic use of medication can be continued but should be registered (for example: analgesics…).

6. METHODS

6.1 Study parameters/endpoints

6.1.1 Main study parameter/endpoint

Evaluation of serum AMH before (baseline) and after (at 3 months follow up) laparoscopic treatment of endometrioma(s):

6.1.2 Secondary study parameters/endpoints

- AMH difference/cyst surface between baseline and at 3, 6 and 12 months post-operative with a correction for the cyst surface (since the volume of the cyst may be responsible for more/less influence on the ovarian reserve)
- AMH modifications at 6 and 12 months follow up
- Cyst recurrence rate at 3, 6, 12 and 24 months postoperatively (visualized by transvaginal ultrasound)
- Clinical pregnancy (as defined by the ICMART (16) as a pregnancy diagnosed by ultrasonographic visualization of one or more gestational sacs or definitive clinical signs of pregnancy. It includes ectopic pregnancy. Note: Multiple gestational sacs are counted as one clinical pregnancy.)
- Ectopic pregnancy (as defined by the ICMART (16) as a pregnancy in which implantation takes place outside the uterine cavity)
- Miscarriage (defined as a spontaneous loss of pregnancy)
- Live birth (as defined by the ICMART (16) as the complete expulsion or extraction from its mother of a product of fertilization, irrespective of the duration of the pregnancy, which, after such separation, breathes or shows any other evidence of life, such as heart beat, umbilical cord pulsation, or definite movement of voluntary muscles, irrespective of whether the umbilical cord has been cut or the placenta is attached.

- Evolution of pain patterns pre- and postoperatively: each endometriosis related pain complaint will be evaluated using the NRS scale at each visit.
- POI postoperatively

6.1.3 Other study parameters

Baseline data:

- age of the participant
- use of contraception + type
- smoking
- previous surgery for ovarian cysts
  - laparoscopy
  - laparotomy
- previous surgery for endometriosis
  - number of surgeries
- indication for surgery:
  - pain
  - Infertility
- pain pattern (+NRS for each parameter)
  - dysmenorrhea
  - chronic pelvic pain/non menstrual pelvic pain
  - dyspareunia
  - dyschezia
  - dysuria
- infertility:
  - duration of infertility
  - primary of secondary infertility
- gravida/para
- BMI (kg/m^2)
- Number of endometriomas
- Diameter of all the cysts (measured in 3 orthogonal planes in mm)
  - Cyst 1
  - Cyst 2
  - …
- Cyst wall surface
- Uni or bilateral endometriomas
- Baseline AMH value

<u>Surgical data:</u>

- Total Operative time (min)
- Operative time for endometrioma (min)
- Hemostatic method used to manage the bleeding on each ovary
  - Not required (no bleeding)
  - Selective bipolar coagulation + hemostatic device used + power settings
  - Suturing
  - Hemostatic sealants
- rASRM points and stage
- EFI
- Hospital stay (days)
- Follow up (months)
- Complications until 3 months postoperatively as defined by the Clavien-Dindo classification (21)

<u>Postoperative follow-up</u>:

- Baseline questionnaire concerning pain pattern (+NRS for each parameter) at 3, 6, 12 and 24 months follow up
  - dysmenorrhea
  - chronic pelvic pain/non menstrual pelvic pain
  - dyspareunia
  - dyschezia
  - dysuria
- Child wish, if present report management in past period (at 3, 6, 12 and 24 months follow up):
  - Non-ART management:
    - Spontaneously +/- ovulation induction
    - IUI
  - ART management:
    - IVF
    - ICSI
    - Egg donation
- Transvaginal ultrasound (at 3, 6, 12 and 24 months follow up)
- AMH value at 3*, 6 and 12 postoperatively (*standard care)

6.2 Randomization, blinding and treatment allocation

Patients fitting the inclusion criteria and consenting to participate are randomly assigned according to a computer-generated randomization list using the method of block randomization (using varying block sizes) to allocate them in proportion, at 1:1, either to Group 1 (combined technique) or Group 2 (CO_2 laser vaporization).

Randomization will be done maximally 2 months before the intervention and minimally 1 hour before start of the intervention by the (sub)investigator of each center. Block randomization per study center will be used to ensure allocation of equal numbers of subjects in each group per center.

Blinding of the surgeon is not possible. Blinding of surgeons/patients during postoperative follow up is not feasible and is assumed to not influence the primary outcome. Indeed, bias due to lack of blinding is expected to be negligible since the primary outcome measured is clear and unambiguous (measurement of AMH).

6.3 Study procedures

6.3.1 Participants flow

STUDY FLOW CHART

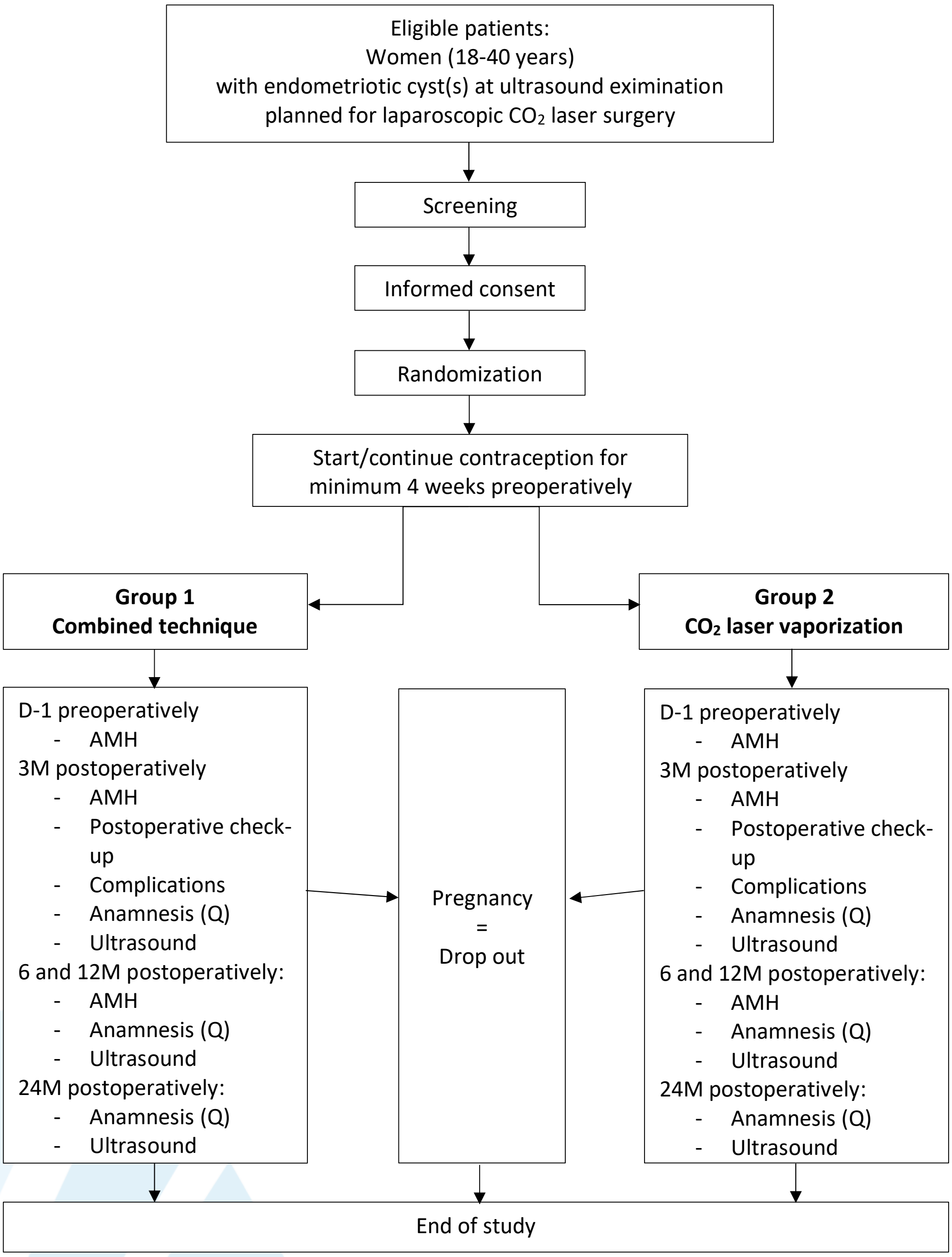

6.3.2 Time requirements

Patient recruitment will start after approval by the ethics committee. Estimated inclusion time of 60 months (initially estimated inclusion time of 24 months, due to the COVID-19 pandemic important delay in recruitment for which extension of inclusion time with another 36 months). Primary outcome will be analyzed at 3 months follow-up postoperatively. Secondary outcomes will be analyzed at 24 months follow-up postoperatively. If necessary, extend with 9 months to allow full follow-up of pregnancies until delivery.

6.3.3 Trial monitoring and patient visits

All patients will be seen by the principal/subinvestigators or by one of his/her staff members at the following moment:

- Screening = standard of care
  Consultation for discussion of the standard preoperative examination, study information will be given if patient is eligible for inclusion.
  AMH value = standard of care for patients planned for laparoscopic surgery for an endometriotic cyst (part of the screening process)
- Baseline = study visit (can be combined with screening)
  Completion of the informed consent forms
- Elective hospitalization for surgery = standard of care
  Randomization
  AMH value = study sample
- 3 months postoperatively (visit window: minimally 3 months postoperatively + 2 weeks)
  Visit with postoperative checkup, complications assessment using the Clavien-Dindo classification, anamnesis concerning pain and child wish and transvaginal ultrasound = standard of care
  AMH value = standard of care
- 6 months postoperatively (+/- 2 weeks)
  Visit with anamnesis concerning pain and child wish and transvaginal ultrasound = standard of care
  AMH value = study sample
- 12 months postoperatively (+/- 2 weeks)
  Visit with anamnesis concerning pain and child wish and transvaginal ultrasound = standard of care

AMH value = study sample

- 24 months postoperatively (+/- 2 weeks)
  Visit with anamnesis concerning pain and child wish and transvaginal ultrasound = standard of care

Procedures	Visits						
	Screening	Baseline*	Hospitalization	Follow up			
				3 months	6 months	12 months	24 months
History/clinical exam	x						
Informed consent		x					
Eligibility assessment		x					
Randomization		x					
Laparoscopic surgery (group 1 vs group 2)			x				
Postoperative check-up				x			
Complication assessment				x			
Anamnesis				x	x	x	x
Ultrasound				x	x	x	x
AMH-value	x		D-1*	x	x*	x*	

* = study sample/visit

Blood samples for AMH measurements (using Roche ECLIA AMH kit): standard of care sample will be done at the laboratory of each participating center – study samples will be sent to the laboratory of UZ Leuven.

6.3.4 Study costs

Blood samples for AMH measurements: total blood samples: 5
Kit used for AMH measurement: Roche ECLIA AMH: cost per analysis: 27€
(2 payed by the patients – 3 for the study = 3x27€ = 81€)
Total cost for AMH measurement based on a sample size of 92 patients = 92x3x27€ = 7452€

Consultations and transvaginal ultrasounds = standard of care.

Organization of the study is done by a trainee in gynecology and obstetrics/PhD student (cost of a PhD student/year: for the full project duration: first year 41343,40€– second year 43140,99€– third year 45179,77€– fourth year 47071,49€). Estimated time requirement per week for this study: 1 day/week.

Help of a study nurse (cost of a study nurse/year: 45000€). Estimated time requirement per week for this study: 0,5 day/week.

Statistical analysis will be done by a statistician (Steffen Fieuws).

6.3.5 Budget and funding

14065€ has been granted by the Fond Academische Studies of the University Hospitals Leuven. Budget for consumables (study related AMH measurements), statistical support and use of the eCRF (Redcap) for the duration of the study.

6.4 Withdrawal of individual subjects

Subjects can leave the study at any time for any reason if they wish to do so without any consequences. The investigator can decide to withdraw a subject from the study for urgent medical reasons. Each withdrawal must be clearly documented.

7. SAFETY REPORTING

Both arms of the study are existing and accepted surgical strategies. The fertility surgeons participating master both techniques equally well.

7.1 Temporary halt for reasons of subject safety

The DSMB can terminate the study if in their opinion a significant number of adverse events have occurred as a result of performing the study. Although unexpected severe adverse events are not anticipated since this trial compares two existing and accepted surgical strategies.

7.2 Definitions AEs, SAEs and SUSARs

7.2.1 Adverse events (AEs)

An AE is any untoward medical occurrence in a patient or subject during an experiment, and which does not necessarily have a causal relationship with the specific surgical intervention. An AE can therefore be any unfavourable and unintended sign (including an abnormal laboratory finding), symptom or disease temporally associated with the use of a specific intervention, whether or not considered related to the intervention. Any worsening (i.e., any clinically significant adverse change in the frequency or intensity of a pre-existing condition) should be considered an AE.

7.2.2 Serious adverse events (SAEs)

A serious adverse event is any untoward medical occurrence or effect that

- results in death;
- is life threatening* (at the time of the event);

- requires hospitalization or prolongation of existing inpatients' hospitalization;
- results in persistent or significant disability or incapacity;
- is a congenital anomaly or birth defect; or
- any other important medical event that did not result in any of the outcomes listed above due to medical or surgical intervention but could have been based upon appropriate judgement by the investigator.

An elective hospital admission will not be considered as a serious adverse event.

** The term "life threatening" in the definition of SAE refers to an event in which the subject was at risk of death at the time of the event. It does not refer to an event which hypothetically might have caused death if it was more severe.*

7.2.3 Suspected Unexpected Serious Adverse Reaction (SUSAR)

A SUSAR is an adverse reaction, the nature or severity of which is not consistent with the information on the experiment, and, when a clinical trial is concerned, with the applicable product information (e.g. the patient leaflet joined to the summary of product characteristics for an authorised product).

7.3 Adverse events that do not require reporting

The following events are considered reportable:

- any SAE*
- reintervention for bleeding on the operated ovary
- POI

*For this study the following serious adverse events are not to be considered as SAE and shoult not be reported to the Sponsor, these should be recorded in the patient's medical notes according to routine practice:

- Pre-planned hospitalizations unless the condition for which the hospitalization was planned has worsened from the first trial-related activity after the subject has signed the informed consent.
- Hospitalization as part of a standard procedure for protocol therapy execution. However, hospitalization or prolonged hospitalization for a complication of the intervention will be reported as an SAE.
- Hospitalization or prolongation of hospitalization for technical, practical, or social reasons, in absence of an AE.
- Treatment of a pre-existing disease that is not aggravated
- Treatment of a pre-existing disease that has not worsened

In general, the following should not be reported as AEs:

- Pre-existing conditions, including those found as a result of screening (these should be reported as medical history or concomitant illness).
- Pre-planned procedures unless the condition for which the procedure was planned has worsened from the first trial-related activity after the subject has signed the informed consent.

7.4 Recording and reporting of Adverse events

Investigators will seek information on AEs during each patient contact. All events, whether reported by the patient or noted by trial staff, will be recorded in the patient's medical record and in the (e)CRF within a reasonable time after becoming aware. If available, the diagnosis should be reported on the AE page, rather than the individual signs or symptoms. If no diagnosis is available, the Investigator should record each sign and symptom as individual AEs.

The following minimum information should be recorded for each AE:

- AE description
- start and stop date of the AE
- severity
- seriousness
- causality assessment to the study interventions
- outcome

7.4.1 Assessment

All AEs must be evaluated by an Investigator as to:

- Seriousness: whether the AE is an SAE. See above for the seriousness criteria.
- Severity: Severity must be evaluated by an Investigator according to the following definitions:
    - Mild – no or transient symptoms, no interference with the subject's daily activities
    - Moderate – marked symptoms, moderate interference with the subject's daily activities
    - Severe – considerable interference with the subject's daily activities, unacceptable
- Causality:
    - None – An AE which is not related to the study-related interventions
    - Unlikely – An AE for which an alternative explanation is more likely (e.g. concomitant medication(s), concomitant disease(s)), and/or the relationship in time suggests that a causal relationship is unlikely
    - Possible – An AE which might be due to the study-related interventions. An alternative explanation is inconclusive. The relationship in time is reasonable; therefore the causal relationship cannot be ruled out.
    - Probable - An AE which might be due to the study-related intervention. The relationship in time is suggestive (e.g. confirmed by dechallenge). An alternative explanation is less likely.
    - Definitely – An AE which is known as a possible adverse reaction and cannot be reasonably explained by an alternative explanation. The

relationship in time is very suggestive (e.g. it is confirmed by dechallenge and rechallenge).

7.4.2 Timeline for reporting

All reportable events will be reported, without undue delay after obtaining knowledge of the events, to the sponsor and chief investigator by mail. This reporting is done by using the appropriate SAE form (in the eCRF). The following information will be recorded: description, date of onset and end date, severity, assessment of relatedness to the specific surgical technique. Follow up information should be provided as necessary.

Contact details for reportable events:

Chief investigator: Prof Dr C. Tomassetti & Dr. C. Bafort: UZ Leuven, Herestraat 49, 3000 Leuven.

Email: carla.tomassetti@uzleuven.be & celine.bafort@uzleuven.be

Sponsor: UZ Leuven – Legal representative: Prof Dr W. Robberecht, Herestraat 49, 3000 Leuven

If an authorized Investigator from the reporting site is unavailable, initial reports without causality and expectedness assessment should be submitted to the Sponsor by a healthcare professional within 24 hours of becoming aware of the SAE, but must be followed-up by medical assessment as soon as possible thereafter.

7.4.3 Follow-up

The Investigator must record follow-up information by updating the medical records and the appropriate forms in the (e)CRF. The worst-case severity and seriousness of an event must be kept throughout the trial.

SAE follow-up information should only include new (e.g. corrections or additional) information and must be reported within 24 hours of the Investigator’s first knowledge of the information. This is also the case for previously non-serious AEs which subsequently become SAEs.

- All SAEs must be followed up until the outcome of the event is ‘recovered’, ‘recovered with sequelae’, ‘not recovered’ (in case of death due to another cause) or ‘death’ (due to the SAE) and until all related queries have been resolved, or until end of trial (whichever occurs first).
- Non-serious AEs must be followed up until the patient’s last study visit, and until all related queries have been resolved.

SAEs after the end of the trial: If the Investigator becomes aware of an SAE with suspected causal relationship to the study-related interventions after the subject has ended the trial, the Investigator should report this SAE within the same timelines as for SAEs during the trial.

All reportable AEs will be followed until they have abated, or until a stable situation has been reached. Depending on the event, follow up may require additional tests or medical procedures as indicated, and/or referral to the general physician or a medical specialist.

7.4.4 Pregnancy

Female subjects must be instructed to notify the Investigator immediately if they become pregnant during the trial. Pregnancy is a secondary outcome in the study, is no (S)AE and should therefore not be reported to the Sponsor.

7.4.5 Death

All deaths will be reported without delay to the Sponsor (irrespective of whether the death is related to the intervention or is an unrelated event). The sponsor will notify all deaths, as soon as possible after becoming aware, to the Central EC and the EC of the concerned site and provide additional information if requested.

Reporting requirements to Ethics Committee's (EC's) The Investigator is responsible for ensuring that all safety events are recorded in the (e)CRF and reported to the Sponsor.

The Sponsor will promptly evaluate all SAEs and AESIs against medical experience to identify and expeditiously communicate possible new safety findings to Investigators and based on applicable legislation.

7.4.6 Annual reporting

The sponsor has the obligation to, once a year throughout the clinical trial (or on request), submit a progress report to the EC's containing an overview of all SARs occurred during the reporting period and taking into account all new available safety information received during the reporting period.

7.4.7 Overview reporting requirements

	WHAT	HOW	TO	TIMELINES
Investigator	AE	AE form	sponsor	as defined in protocol
	SAE	SAE form	sponsor	Immediately (within 24 hours of becoming aware of the event)<br>Exceptions: as defined in protocol

	death	SAE form	sponsor	asap
Sponsor	death	SAE form + narrative	Ethics Committees	asap
	Annual Progress Report	APR template	Ethics Committees	annually

7.5 Data Safety Monitoring Board (DSMB)

Following person(s) will be part of the DSMB:

- independent statistician of the KULeuven (no conflict of interest concerning this trial).

The advice(s) of the DSMB will only be sent to the sponsor of the study. Should the sponsor decide not to fully implement the advice of the DSMB, the sponsor will send the advice to the reviewing METC, including a note to substantiate why (part of) the advice of the DSMB will not be followed.

8. STATISTICAL ANALYSIS

8.1 Sample size calculation:

Sample size calculation is based on the primary outcome: evaluation of serum AMH 3 months after the specific surgical treatment.

Power calculation of this study was based on the findings of the RCT from Candiani et al (8) where AMH was a secondary outcome in comparing conventional cystectomy versus CO2 laser vaporization. In this paper a postoperative AMH of 1,9 ± 0,9 ng/mL was found after CO_2 laser vaporization only. In this paper a difference in decline in postoperative AMH of 50% was observed in favor of the vaporization group, although this study was not powered for this outcome. Since we will compare two conservative techniques for endometrioma surgery, a difference of 30% AMH decline after surgery was considered to be clinically relevant (after consultation of all participating centers). Based on Candiani et al. (2018) a mean serum AMH of 1.9 (SD=0.9) ng/mL is expected with CO2 laser vaporization. Assuming a common standard deviation a total sample size of 82 patients is needed (or 41 patients in each group) based on a two-sided independent t-test with alpha equal to 0.05 to have at least 80% power to detect a difference of 30% between both groups. Note that this calculation is based on the conservative assumption of no correlation between baseline AMH and AMH after 3 months.

In practice, the power is expected to exceed largely 80% since the final analysis will be based on an ANCOVA approach. However, since the study wants to gather information on multiple endpoints, we deemed it not appropriate to lower the sampe size.

To account for the 10% drop out because of pregnancy within 3 months postoperatively, it is prudent to aim for a total sample size of 92 patients (or 46 patients in each group).

8.2 Baseline data:

Age of the patient	Continuous
Use of contraception	Categorical
Smoking	Dichotomous
Previous surgery for ovarian cysts<br>- Number of surgeries<br>- Laparoscopy<br>- Laparotomy	Dichotomous<br>Continuous<br>Dichotomous<br>Dichotomous
Indication for surgery: pain and/or infertility	Categorical
Pain pattern (+NRS for each parameter):<br>- Dysmenorrhea<br>- Chronic pelvic pain<br>- Dyspareunia<br>- Dyschezia<br>- Dysuria	Continuous
Infertility<br>- Duration<br>- Primary or secondary	 Continuous<br>Dichotomous
Gravida/ Para	Categorical
BMI (kg/m^2)	Continuous
Diameter of the cysts (in mm)	Continuous
Cyst wall surface	Continuous
Uni or bilateral endometrioma	Dichotomous
Baseline AMH value	Continuous

Surgical data:

Total operative time (min)	Continuous
Operative time for endometrioma (min)	Continuous

Hemostatic method used to manage the bleeding on each ovary<br>- Not required (no bleeding)<br>- Selective bipolar coagulation + hemostatic device used + power settings<br>- Suturing<br>- Hemostatic sealants	Categorical
rAFS points	Continuous
rAFS stage	Ordinal
EFI	Ordinal
Hospital stay (days)	Continuous
Follow up postoperativeley (months)	Continuous
Complications:<br>- Clavien-Dindo grade I<br>- Clavien-Dindo grade II<br>- Clavien-Dindo grade III<br>- Clavien-Dinco grade IV	Ordinal

Postoperative follow-up

Pain pattern (+NRS for each parameter):<br>- Dysmenorrhea<br>- Chronic pelvic pain<br>- Dyspareunia<br>- Dyschezia<br>- Dysuria	Continuous
Recurrence at ultrasound	Dichotomous
AMH value	Continuous
Child wish<br>- Non-ART<br>o Spontaneous<br>o IUI<br>- ART<br>o IVF<br>o ICSI	Dichotomous

Summary tables (descriptive statistics and/or frequency tables) will provided for all variables (baseline, surgical and postoperative follow-up).
Continuous variables will be summarized with descriptive statistics (n, mean, standard deviation, range, median, p25 and p75).
Frequency counts and proportions of subjects within each category will be provided for the categorical data.

Procedure(s) to account for missing data:
Since patients will be randomized after baseline measurements, baseline measurements are available for all randomized patients. We will avoid missing data by using a e-CRF that can only be saved if all necessary data is correctly filled out. Further, in the statistical analysis a modeling approach (direct likelihood approach for the cLDA model on longitudinal data) will be used incorporating all available information, i.e. also subjects with one or more timepoints with a missing outcome are still included in the analysis.

8.3 Primary study parameter(s)

Evaluation of serum AMH at 3 months follow up.
It concerns an intention to treat analysis. All participants will be included in the analysis in the groups to which they were originally assigned, regardless of what subsequently occurred.
To handle the presence of missing values (due to dropout or to pregnancy, in the latter case AMH values after pregnancy are put on missing), a constrained longitudinal data analysis (cLDA, cfr Liang and Zeger, 2010) will be used instead of an ANCOVA to evaluate the differences between the groups in the final analysis. In this approach both the baseline and post-baseline values are modeled as dependent variables, as opposed to a longitudinal ANCOVA model in which the baseline value is included as a covariate. Although the baseline measure is included in the response vector in cLDA, the true baseline means are constrained to be the same for different treatment groups due to randomization, and this analysis provides an adjustment for the observed baseline difference in estimating the treatment effects. Center will be added as a factor in the model. A (logarithmic) transformation of the AMH will be considered to obtain a more symmetric distribution of the model residuals. Using a direct likelihood approach for the cLDA model, a subject with a missing measurement at three months is not excluded from the analysis. As such, the analysis does not assume missing completely at random (MCAR), but is still valid under missing at random (MAR).

References: Liang, K. Y. and Zeger, S. (2000). Longitudinal data analysis of continuous and discrete responses for pre-post designs. Sankhya: The Indian Journal of Statistics, Series B 62, 134–148.

8.4 Secondary study parameter(s)

AMH at other timepoinst

The cLDA model used for the AMH at 3 months will be extended with the other timepoints for the evaluation of the AMH at 6 and 12 months.

AMH /cyst surface at 3, 6 and 12 months follow up

The ratio of the AMH and the cyst surface will be analyzed using the same methodology as used for the AMH.

Cyst recurrence rate

Cumulative recurrences until 24 months postoperatively will be visualised using Kaplan-Meier estimates and compared using stratified log-rank tests (stratified on centre).

Clinical pregnancy, ectopic pregnancy, miscarriage and live birth

This will be analyzed at 24 months follow-up postoperatively. If necessary, extend with 9 months to allow full follow-up of pregnancies until delivery.
Kaplan Kaplan-Meier estimates will be used and comparisons will be based on stratified log-rank tests.

Evolution of pain patterns pre- and postoperatively

Pain scores preoperatively versus 3, 6, 12 and 24 months postoperatively. ll endometriosis related complaints will be questioned and scored using the NRS scale.
The same methology as used for the AMH will be applied to compare the differences in the longitudinally gathered NRS scores.

8.5 Subgroup analysis

Subgroup analysis will be performed on patients:
- Without previous history of ovarian surgery
- Depending on continuation of contraception postoperatively

8.6 Timing of data analysis

Analysis on the primary outcome will be performed when all included patients had their first postoperative blood sample of AMH (3 months postoperatively).

All other analyses after 24 months of follow up (recurrence rate, pregnancy rate,…), if necessary extend with 9 months to allow full follow-up of pregnancies until delivery.

9. ETHICAL CONSIDERATIONS

9.1 Regulation statement

The study will be conducted according to the principles of the Declaration of Helsinki (version 2013: www.wma.net) and in accordance with the Medical Research Involving Human Subjects Act (WMO).

9.2 Recruitment and consent

Recruitment

Women with endometriotic cysts planned for surgical laparoscopic treatment are eligible for the study. Screening for endometriotic cysts will be done using transvaginal ultrasound examination (standard of care). Further preoperative mapping was done according to the standard practice at each center. Eligible women are invited to participate in the study. Eligible women can enter the study only after giving written informed consent. We will not collect data from eligible women who decline to give written informed consent for the study.

Patient identification

Eligible women will be identified by a clinician of the endometriosis team responsible for daily care at the participating centers. We will provide a summary in Layman terms explaining the objectives and the course of the study. Screening for endometriomas and associated DE will be done by a member of the clinical team with expertise in transvaginal ultrasound.

Screening

Transvaginal ultrasound is part of the standard clinical care given to patients with suspected endometriosis. Maximum interval of 6 months between screening and recruitment.

Consent

Written informed consent of capable eligible women will be obtained prior to the randomization after informing the potential participant about the study. The principal investigator at each participating center will retain the overall responsibility for the informed consent of participants at their site. Informed consent will be obtained by the physicians working at the endometriosis clinic of the participating centers. All patients will receive a patient information letter (providing a plain language text in Dutch, French or English) with an informed participant consent form both approved by the central and local EC. During the

process of informed consent, all participants will be given the opportunity to ask questions in their native language.

The right of a participant to refuse participation without giving reasons will be respected under all circumstances. The participant has the legal right to withdraw at any time from the study even after randomization and treatment without giving reasons and without prejudicing her further treatment.

9.3 Benefits and risks assessment, group relatedness

Not applicable

9.4 Compensation for injury

In accordance with the Belgian law of May 7, 2004 regarding experiments on the human person, Institution shall assume, even without fault, the responsibility of any damages incurred by a Study Patient and linked directly or indirectly to the participation to the Study, and shall provide compensation therefore through its insurance.

9.5 Incentives

Not applicable

10. ADMINISTRATIVE ASPECTS, MONITORING AND PUBLICATION

10.1 Handling and storage of data and documents

<u>Source documents</u>

The data will consist of

- Medical history of the patient from the patient’s medical file
- Surgical information from the patient’s medical file
- Data collected during screening/baseline visit, treatment phase and the follow-up period
- Images from the laparoscopy are optional

<u>Electronic case report form (e-CRF)</u>

This study will use an electronic data capture system, i.e. RedCap, for completion of CRFs. The e-CRFs should be fully completed within reasonable time after the patient’s visit. The investigator must verify that all data entries in the e-CRF are accurate. If a patient withdraws from the study, the reason must be noted in the e-CRF.

Data handling and record keeping

All data will be handled, and all record will be kept in accordance with the Belgian law of 30 July 2018 on the protection of individuals with regard to the processing of personal data and the European General Data Protection Regulation 2016/679 (“GDPR”).

RedCap is a web-based system, all study sites will have access to RedCap. The server is hosted within UZ Leuven and meets hospital level security and back-up. Site access will be controlled, login in RedCap is password controlled. Each user will receive a personal login name and password and will have a specific role which had predefined restrictions on what is allowed in RedCap. This entails that only site staff with a specific role can add or change data in RedCap. Users will only be able to see data of patients of their own site. Any activity in this software is traced and transparent per audit trail and log files.

Patients that are included in the study, will be randomized in RedCap. A unique study number will be assigned to all subjects. The subject identification code will be safeguarded by the site. The name and any other identifying data will not be included in the study database.

Data analysis will be performed by a statistician.

Archiving

Preservation of data during minimally 20 years after termination of the trial. At that moment, it will be judged whether it is necessary to retain them for a longer period.

10.2 Monitoring and Quality Assurance

Audit and monitoring are not necessary as both arms of the study represent routinely accepted surgical treatments for endometriomas. The fertility surgeons participating master both techniques equally well.

10.3 Public disclosure and publication policy

The results will be published in scientific journals and all participating investigators are co-authors, according to the number of patients included and intellectual contribution.

11. REFERENCES

1. Burney RO, Giudice LC. Pathogenesis and pathophysiology of endometriosis. Fertility and Sterility. 2012;98(3):511-9.

2. Nisolle M, Donnez J. Peritoneal endometriosis, ovarian endometriosis, and adenomyotic nodules of the rectovaginal septum are three different entities. Fertility and Sterility. 1997;68(4):585-96.
3. Donnez J, Nisolle M, Gillet N, Smets M, Bassil S, Casanasroux F. Large ovarian endometriomas. Hum Reprod. 1996;11(3):641-6.
4. Donnez J, Lousse J-C, Jadoul P, Donnez O, Squifflet J. Laparoscopic management of endometriomas using a combined technique of excisional (cystectomy) and ablative surgery. Fertility and sterility. 2010;94(1):28.
5. Alborzi S, Momtahan M, Parsanezhad ME, Dehbashi S, Zolghadri J, Alborzi S. A prospective, randomized study comparing laparoscopic ovarian cystectomy versus fenestration and coagulation in patients with endometriomas. Fertility and Sterility. 2004;82(6):1633-7.
6. Beretta P, Franchi M, Ghezzi F, Busacca M, Zupi E, Bolis P. Randomized clinical trial of two laparoscopic treatments of endometriomas: cystectomy versus drainage and coagulation. Fertility and Sterility. 1998;70(6):1176-80.
7. Hart RJ, Hickey M, Maouris P, Buckett W. Excisional surgery versus ablative surgery for ovarian endometriomata. Cochrane Menstrual Disorders and Subfertility Group. 2005(3).
8. Candiani M, Ottolina J, Posadzka E, Ferrari S, Castellano LM, Tandoi I, et al. Assessment of ovarian reserve after cystectomy versus 'one-step' laser vaporization in the treatment of ovarian endometrioma: a small randomized clinical trial. Human reproduction (Oxford, England). 2018.
9. Carmona F, Martínez-Zamora MA, Rabanal A, Martínez-Román S, Balasch J. Ovarian cystectomy versus laser vaporization in the treatment of ovarian endometriomas: a randomized clinical trial with a five-year follow-up. Fertility and Sterility. 2011;96(1):251-4.
10. Lambert-Messerlian G, Plante B, Eklund EE, Raker C, Moore RG. Levels of antimüllerian hormone in serum during the normal menstrual cycle. Fertility and sterility. 2016;105(1):208.
11. Depmann M, Eijkemans MJC, Broer SL, Scheffer GJ, van Rooij IAJ, Laven JSE, et al. Does anti-Müllerian hormone predict menopause in the general population? Results of a prospective ongoing cohort study. Human reproduction (Oxford, England). 2016;31(7):1579.
12. Dólleman MM, Verschuren CWM, Eijkemans TMJ, Dollé MME, Jansen MEHJ, Broekmans TFJ, et al. Reproductive and Lifestyle Determinants of Anti-Müllerian Hormone in a Large Population-based Study. The Journal of Clinical Endocrinology & Metabolism. 2013;98(5):2106-15.
13. Su IH, Maas MK, Sluss JP, Chang ER, Hall EJ, Joffe EH. The Impact of Depot GnRH Agonist on AMH Levels in Healthy Reproductive-Aged Women. The Journal of Clinical Endocrinology & Metabolism. 2013;98(12):E1961-E6.
14. Dunselman GAJ, Vermeulen N, Becker C, Calhaz - Jorge C, D'Hooghe T, De Bie B, et al. ESHRE guideline: management of women with endometriosis †. Human Reproduction. 2014;29(3):400-12.
15. Chang HJ, Han SH, Lee JR, Jee BC, Lee BI, Suh CS, et al. Impact of laparoscopic cystectomy on ovarian reserve: serial changes of serum anti-Müllerian hormone levels. Fertility and Sterility. 2010;94(1):343-9.
16. Zegers-Hochschild F, Adamson GD, de Mouzon J, Ishihara O, Mansour R, Nygren K, et al. International Committee for Monitoring Assisted Reproductive Technology (ICMART) and the World Health Organization (WHO) revised glossary of ART terminology, 2009. 2009. p. 1520-4.
17. Van Holsbeke C, Van Calster B, Guerriero S, Savelli L, Paladini D, Lissoni AA, et al. Endometriomas: their ultrasound characteristics. Ultrasound in Obstetrics and Gynecology. 2010;35(6):730-40.
18. Revelli A, Biasoni V, Gennarelli G, Canosa S, Dalmasso P, Benedetto C. IVF results in patients with very low serum AMH are significantly affected by chronological age. An Official Journal of the American Society for Reproductive Medicine. 2016;33(5):603-9.
19. Saridogan E, Becker C, Feki A, Grimbizis G, Hummelshoj L, Keckstein J, et al. Recommendations for the surgical treatment of endometriosis—part 1: ovarian endometrioma. Endoscopic Imaging and Allied Techniques. 2017;14(1):1-7.

20. Vanhie A, Meuleman C, Tomassetti C, Timmerman D, D'Hoore A, Wolthuis A, et al. Consensus on Recording Deep Endometriosis Surgery: the CORDES statement. Human reproduction (Oxford, England). 2016;31(11):2660.
21. Dindo D, Demartines N, Clavien P-A. Classification of Surgical Complications: A New Proposal With Evaluation in a Cohort of 6336 Patients and Results of a Survey. Annals of Surgery. 2004;240(2):205-13.

12. ANNEX: DATA PROCESSING AGREEMENT (“DPA”)

This data processing agreement, including any annexes hereto, (together the "Data Processing Agreement" or “DPA”) is an integrated part of the Protocol.

All defined terms within the Protocol shall have the same meaning when used in this Data Processing Agreement, unless explicitly defined otherwise in this Data Processing Agreement.

12.1 SCOPE OF THE DATA PROCESSING AGREEMENT

The PARTICIPATING SITE acts as a data processor as defined under article 4, 8) of the GDPR (“Data Processor”) for the SPONSOR who acts as data controller as defined under article 4, 7) of the GDPR (“Data Controller”), when the PARTICIPATING SITE processes Personal Data for the Sponsor as set out in Annex 1.

12.2 PROCESSING OF PERSONAL DATA

12.2.1 Instructions**: The Data Processor is instructed to process the Personal Data for the term of this Data Processing Agreement and only for the purposes of providing the data processing tasks set out in Annex 1. The Data Processor may not process or use Personal Data for any purpose other than a Data Subject’s medical records or other than provided in the Agreement or instructions, including with regard to transfers of personal data to a third country or an international organization, unless the Data Processor is required to do so according to Union or Member State law. In that case, the Data Processor shall inform the Data Controller in writing of that legal requirement before processing, unless that law prohibits such information on important grounds of public interest.**

12.2.2 Data Processor shall at all times maintain a record of processing of Personal Data in accordance with Applicable Law and if the Data Processor considers an instruction from the Data Controller to be in violation of the Applicable Law, the Data Processor shall promptly inform the Data Controller in writing about this.

12.3 THE DATA PROCESSOR'S OBLIGATION

12.3.1 The Data Processor must ensure that persons authorized to process the Personal Data have committed themselves to confidentiality or are under an appropriate statutory obligation of confidentiality. The Data Processor shall take full responsibility in the event there is a breach of said confidentiality obligation.

12.3.2 The Data Processor shall implement appropriate technical and organizational measures to prevent that the Personal Data processed is:

- accidentally or unlawfully destroyed, lost or altered,
- disclosed or made available without authorization, or
- otherwise processed in violation of Applicable Law.

12.3.3 The Data Processor must also comply with the special data security requirements of Annex 1.

12.3.4 The appropriate technical and organizational security measures must be determined with due regard for:

- the current state of the art,
- the cost of their implementation, and
- the nature, scope, context and purposes of processing as well as the risk of varying likelihood and severity for the rights and freedoms of natural persons.

12.3.5 The Data Processor shall upon request provide the Data Controller with sufficient information to enable the Data Controller to ensure that the Data Processor's obligations under this Data Processing Agreement are complied with, including ensuring that the appropriate technical and organizational security measures have been implemented.

12.3.6 The relationship of the Parties and the nature of the Study outlined in the Agreement are such that the Data Controller has no access to the identity of the Study Participants. Therefore, the Data Controller needs to rely on the Data Processor in order to be able , by means of appropriate technical and organizational measures, to fulfil the obligation imposed to the Data Controller under Applicable Laws. Data Processor shall therefore respond to requests from Data Subjects in accordance with Article Error! Reference source not found. **of the Agreement pursuant to Applicable Laws (such as, the right of access, the right to rectification, the right to erasure, the right to restrict the processing, the right to data portability and the right to object).**

12.3.7 The Data Controller is entitled to appoint at its own cost an independent expert, who shall have access to the Data Processor's data processing facilities and receive the necessary information for the sole purpose of auditing whether the Data Processor has complied with its obligations The Data Processor may reasonably and in a justified manner object to the appointment of this proposed expert. The expert shall upon the Data Processor's request sign a non-disclosure agreement provided by the Data Processor, and treat all information obtained or received from the Data Processor confidentially, and may only pass on, the findings as described under clause - below to the Data Controller.

12.3.8 The Data Processor must give authorities who by Union or Member State law have a right to enter the Data Controller's or the Data Controller's processors' facilities, or representatives of the authorities, access to the Data Processor's

physical facilities against proper proof of identity and mandate, during normal business hours and upon reasonable prior written notice.

12.3.9 The Data Processor must without undue delay, and where feasible within 72 hours, notify the Data Controller in writing about:

- any request for disclosure of Personal Data processed under the Agreement by authorities, unless expressly prohibited under Union or Member State law
- any finding of (a) breach of security that results in accidental or unlawful destruction, loss, alteration, unauthorized disclosure of, or access to, Personal Data transmitted, stored or otherwise processed by the Data Processor under the Agreement (“Data Breach”), or (b) other failure to comply with the Data Processor's obligations under Clause 12.3, or
- any request for access to the Personal Data (with the exception of medical records of the Study Participants for which the Data Processor is considered Data Controller) received directly from the Data Subjects or from third parties.

12.3.10 Such a notification from the Data Processor to the Data Controller with regard to a breach of security as meant in Clause -(a) will contain at least the following information:

- The nature of the Personal Data Breach, stating the categories and (by approximation) the number of Data Subjects concerned, and stating the categories and (by approximation) the number of the personal data registers affected (datasets);
- The likely consequences of the Personal Data Breach;
- A proposal for measures to be taken to address the Personal Data Breach, including (where appropriate) measures to mitigate any possible adverse effects of such breach.

The Data Processor shall document (and shall keep such documentation available for the Data Controller) any Personal Data Breaches, including the facts related to the Personal Data Breach, its effects and the corrective measures taken. After consulting with the Data Controller, the Data Processor shall take any measures needed to limit the (possible) adverse effects of Personal Data Breaches (unless such consultation cannot be awaited due to the nature of the Personal Data Breach).

12.3.11 The Data Processor must promptly and reasonably execute all actions required to handle (a) responses to any breach of security as described in - above and (b) any requests from Data Subjects under Chapter III of the GDPR, including requests for access, rectification, restriction of processing or erasure. The Data Processor must also reasonably implement the appropriate technical and organizational measures to enable the Data Controller to fulfil the Data Controller's obligation to respond to such requests. Any reasonable documented costs and expenses pre-approved in writing by the Data Controller related to the above will be reimbursed by the Data Controller to the extent such costs and expenses are not related to any requirements according to Applicable Law imposed on the Data Processor or due to any breach of this DPA or the Protocol by Data Processor.

12.3.12 The Data Processor must reasonably assist the Data Controller with meeting the other obligations that may be incumbent on the Data Controller

according to Union or Member State law where the assistance of the Data Processor is implied, and where the assistance of the Data Processor is necessary for the Data Controller to comply with its obligations. This includes, but is not limited to, at the request to provide the Data Controller with all necessary information about an incident under Clause -, and all necessary information for an impact assessment in accordance with Article 35 and Article 36 of the GDPR. Any reasonable documented costs and expenses pre-approved in writing by the Data Controller related to the above will be reimbursed by the Data Controller to the extent such expenses are not related to any requirements according to Applicable Law imposed on the Data Processor or due to breach of this DPA or the Protocol by Data Processor.

12.4 SUBPROCESSORS

12.4.1 The Data Processor may only engage a subprocessor, with prior specific or general written consent from the Data Controller. At the time of this Data Processing Agreement, the Data Processor uses the subprocessor listed in Annex 2. The Data Processor undertakes to inform the Data Controller of any intended changes concerning the addition or replacement of a subprocessor by providing a reasonable prior written notice to the Data Controller. The Data Controller may reasonably and in a justified manner object to the use of a subprocessor. The Data Processor must inform the Data Controller in writing of the discontinued use of a subprocessor.

12.4.2 Prior to the engagement of a subprocessor, the Data Processor shall conclude a written agreement with the subprocessor, in which at least the same data protection obligations as set out in this Data Processing Agreement shall be imposed on the subprocessor, including obligations to implement appropriate technical and organizational measures and to ensure that the transfer of Personal Data is done in such a manner that the processing will meet the requirements of the Applicable Law.

12.4.3 T**he Data Controller has the right to receive a copy of the relevant provisions of Data Processor's agreement with the subprocessor related to data protection obligations. The Data Processor shall remain fully liable to the Data Controller for the performance of the subprocessor obligations under this Data Processing Agreement. The fact that the Data Controller has given consent to the Data Processor's use of a subprocessor is without prejudice for the Data Processor's duty to comply with this Data Processing Agreement.**

12.5 CONFIDENTIALITY

12.5.1 The Data Processor shall keep Personal Data confidential.

12.5.2 The Data Processor shall not disclose the Personal Data to third parties or take copies of Personal Data unless strictly necessary for the performance of the Data Processor's obligations towards the Data Controller according to this Data Processing Agreement, and on condition that whoever Personal Data is disclosed to is under the responsibility of a professional subject to the obligation of professional secrecy under Union or Member State law or rules established by national competent bodies or by another person also subject to an obligation of secrecy under Union or Member State law or rules established by national competent bodies.

12.5.3 The Data Processor shall ensure that all employees and any persons that it involves in the conduct of the Study comply with this Data Processing Agreement.

12.5.4 The Data Processor shall limit the access to Personal Data to all employees and any persons that it involves in the conduct of the Study for whom access to said data is necessary to fulfil the Data Processor's obligations towards the Data Controller.

12.5.5 The obligations of the Data Processor under Clause 5 shall continue until such time as provided by Applicable Law and regardless of whether the cooperation of the parties has been terminated.

12.6 TERM AND TERMINATION OF THE DATA PROCESSING AGREEMENT

12.6.1 Regardless of the expiry or termination, for whatever reason, of the Agreement, this Data Processing Agreement r**emains in force and applicable as long as the Data Processor processes the Personal Data for the Data Controller under the Agreement.**

12.6.2 In case of termination of the Agreement, the Data Processor must provide the necessary transition services to the Data Controller. The Data Processor is obliged to reasonably assist Data Controller at Data Controller’s expense.

Data Processor shall have appropriate procedures in place for the archiving of the Personal Data after the end of the Study in accordance with Applicable Law and at the end of the legally mandated archiving period ensure the destruction of the Personal Data and promptly inform Data Controller of this same.

12.6.3 If the Data Processor is required based on Union or Member State law to retain all or part of the Personal Data for a longer period than is possible based on the period mentioned in the Data Processing Agreement, the Data Processor shall immediately communicate this to the Data Controller, stating the basis, term and scope of such obligation. Once compliance with the obligation is no longer impeded by Union or Member State law, the Data Processor shall as yet erase the data in accordance with the provisions in the Data Processing Agreement.

Annexes:

Annex 1: Instructions

Annex 2: Subprocessors

Annex 1 – Instructions

This Annex 1 constitutes the Data Controller's instruction to the Data Processor in connection with the Data Processor's Personal Data processing for the Data Controller and is an integrated part of the Data Processing Agreement.

Contact details of the Data Controller (including its Data Protection Officer, if applicable):
Gdprc.research@uzleuven.be
Contact details of the Data Processor (including its Data Protection Officer, if applicable): /

The processing of Personal Data

a) Purpose and nature of the processing operations
   - Performance of Clinical Study services under the Agreement and for the purpose of mandatory safety monitoring– as specifically described in the Protocol.
   - Completion of data in the CRF system

   I. Transfer of Personal Data to a third country: ~~YES~~/NO
   II. If YES to I., transfer outside the EU: N/A
   III. If YES to II.: N/A

b) Categories of Data Subjects
   I. Former, current or future persons and/or patients who voluntarily enrolled in the Study

c) Categories of Personal Data
   Re b) I: Date of birth and/or age, initials, personal identification number assigned to Data Subjects participating in the Study, description of characteristics of physical features of the body, medical condition, medical images and scans (such as X-ray and study results), drugs and other treatments administered during the Study
   Re b) II: Specific surgical intervention

d) Special categories of Personal Data
   Re b) I: Health information including past medical history, medical condition and its development during the Study, medical test information (such as blood samples results from scans and biopsies) generated during the Study, treatment administered in the course of the Study, data revealing racial or ethnic origin, genetic data and/or social security number

e) Multicenter study: 4 study locations:
   i. UZ Leuven
      Herestraat 49
      3000 Leuven – Belgium
   ii. CHR La Citadelle
      Boulevard du Douzième de Ligne 1
      4000 Liège – Belgium
   iii. GZA Sint-Augustinus
      Oosterveldlaan 24
      2610 Antwerpen – Belgium
   iv. Cliniques universitaires Saint-Luc / UCL
      Avenue Hippocrate 10
      1200 Bruxelles – Belgium

f) Specific security requirements

The following requirements reflect the minimum data processing requirements expected of the Data Processor. It is a condition that other agreed documents, legislation or industry standards laying down requirements of the processing of Personal Data in connection with Study/ /mandatory safety monitoring are complied with as well.

1. The Personal Data may only be used for the Study and/or mandatory safety monitoring.
2. The collection, registration and other processing of Personal Data must be legally authorized under Applicable Law, or applicable policies issued of the supervisory authorities.
3. Any person who takes part in the processing of Personal Data must be familiar with these requirements.
4. Premises used for the storage and other processing of Personal Data must be arranged in such a way as to prevent unauthorized access.
5. Appropriate security measures must be implemented to protect data against accidental or unlawful destruction, loss or impairment. Furthermore, it must be ensured that no incorrect or misleading Personal Data is processed. Incorrect or misleading data, or data processed in contravention of the above Applicable Law, policy of the supervisory authority or these requirements, shall be rectified or erased.
6. Personal Data may not be stored in a way that makes it possible to identify the Data Subjects for longer than is necessary for the achievement of the Study and/or mandatory safety monitoring.
7. The publication of results from clinical studies must take place in such a way that it is impossible to identify individual persons.
8. It is a condition that other legislation laying down requirements of the processing of Personal Data in connection with Study and/or mandatory safety monitoring is complied with.

Electronic data

9. Identification data must be encrypted or replaced by a code number or similar. Alternatively, all data stored can be encrypted. Encryption keys, code keys, etc. must be stored securely and separately from the Personal Data. This also applies to Personal Data that is stored on portable devices such as laptop PCs, tablets, etc.
10. Data may only be accessed by using a unique user name and a confidential password. The password must be renewed at least once a year and when otherwise necessary in order to ensure the secure processing of the data.
11. On the transfer of Personal Data via the internet or other external networks, the necessary security measures must be taken to ensure that the Personal Data does not come to the knowledge of any unauthorized persons. This includes that encryption is required if sensitive Personal Data is transferred via the internet (or other open networks), and security of authenticity (identities of transmitter and recipient) and integrity (the authenticity of the transmitted Personal Data) must be appropriately ensured by the use of suitable security measures. On using internal networks, it must be ensured that no unauthorized persons can gain access to the data.
12. Removable storage media, safety copies of Personal Data, etc. must be stored securely and under lock and key, so that unauthorized access is prevented.

Manual ("paper") data

13. Manual material, including print-outs, error and control lists, etc. with Personal Data, must be stored securely under lock and key, and in such a way as to prevent unauthorized access.

Biobank and biological material

14. N/A

Information to be given to the Study Participant / Data Subject

15. Where the Personal Data is obtained from the Study Participant/ Data Subject (via interviews, questionnaires, clinical or para-clinical examination, treatment, observation, etc.), more detailed information concerning the clinical Study/testing/safety monitoring shall be distributed/forwarded to the Data Subject in accordance with Article 13 of the GDPR. The Study Participant must, via the privacy notice or via the informed consent form (as applicable) as drafted by the Data Controller and as approved by the relevant ethics committee and /or relevant authorities, be informed of the name of the Data Controller and of the name of the Data Processor with clear indication that the Data Processor shall act as the first point of contact with the Study Participant in connection to the processing of the Personal Data and/or with the exercise of rights granted to Data Subjects under the GDPR, the purpose of the trial/testing/safety monitoring, the fact that it is voluntary to participate in the trial/testing, the identity of any recipients of Personal Data, and the purpose of the disclosure of Personal Data, as well as any further information which is necessary for the Study Participant / Data Subject to be able to safeguard his/her interests. The Data Subject has been informed about the right of access to the Personal data that is processed concerning the person in question.

Disclosure

16. Disclosure/issue of Personal Data to other parties may take place to the extent that this is legally authorized under Applicable Law.

On the conclusion of the project

17. At the latest on the conclusion of the Study/testing/safety monitoring the Personal Data (including biological material) shall be erased, made anonymous, or destroyed, unless Union or Member State law requires continued storage of the Personal Data. In accordance with Belgian Law as defined in the Agreement the Data Processor shall be allowed to store the medical records for at least 30 years. It must not subsequently be possible to identify individuals participating in the clinical Study/testing/safety monitoring. The deletion of Personal Data must be properly documented.

18. Alternatively, the Personal Data may be transferred for further storage in archives according to the Data Controller's instructions. Any costs related to such transfer and further storage of Personal Data shall be borne by the Data Controller.

19. Erasure of Personal Data from electronic media shall take place in such a manner that it is impossible to recover the Personal Data and such erasure must be properly documented.

Annex 2 – Subprocessors

The Data Controller agrees that the Data Processor – subject to compliance with Clause 4 of the Data Processing Agreement – engages the parties listed below as subprocessors.

None
