# Supplementary material for: Conservative endometrioma surgery: The combined technique versus CO2-laser vaporization only (BLAST: Belgium LAser STudy): Clinical protocol for a multicenter randomized controlled trial
Source: PLoS One. 2025 Mar 6;20(3):e0315709. doi: 10.1371/journal.pone.0315709 (PMC11884717; doi:10.1371/journal.pone.0315709)
Supplement: S3 File — (PDF) [file pone.0315709.s003.pdf]

Title of the study: BLAST: A randomized controlled trial on conservative endometrioma surgery using the CO2 laser : the combined technique versus CO2 laser vaporization only.

Sponsor of the study: UZ Leuven – Herestraat 49 – 3000 Leuven

Medical Ethics Committee: Ethische commissie onderzoek UZ/KU Leuven (EC onderzoek)

Local investigators: ……………………………………

I Information vital to your decision to take part

Introduction

You are being invited to take part in a clinical study to compare two surgical techniques for the treatment of endometrioma(s) and their effect on the ovarian reserve. A laparoscopic surgery will be performed to remove all the visible endometriosis and restore the normal anatomic proportions as much as possible.

The sponsor and investigator hope that this study may offer advantages in the treatment of patients with the same disease as yours. There is, however, no guarantee that you will benefit from taking part in this study.

Before you agree to take part in this study, we invite you to take note of its implications in terms of organisation, possible risks and benefits, to allow you to make a decision with full awareness of the implications. This is known as giving “informed consent”.

Please read these few pages of information carefully and ask any questions you want to the investigator or his/her representative. There are 3 parts to this document: the information essential to your decision, your written consent and supplementary information (appendices) detailing certain aspects of the basic information.

If you take part in this clinical study, you should be aware that:

- This clinical study is being conducted after having been reviewed by one or more ethics committees.
- Your participation is voluntary and must remain free from any coercion. It requires the signature of a document expressing your consent. Even after having signed this document, you can stop taking part by informing the investigator. Your decision not to take part or to stop taking part in the study will have no impact on the quality of your care or on your relationship with the investigator.
- The data collected on this occasion are confidential and your anonymity is guaranteed during publication of the results.
- Insurance has been taken out in case you should suffer any damage in connection with your participation in this clinical study.
- You will not incur any charges for the visits/consultations, examinations or treatments specific to this study.
- You may contact the investigator or a member of his/her team at any time should you need any additional information.

Further information about your “Rights as a participant in a clinical study” can be found in the appendix.

UZ LEUVEN GYNAECOLOGIE EN VERLOSKUNDE
Leuvens universitair fertiliteitscentrum

Objectives and description of the study protocol

Endometriosis is a gynecological disorder defined by the presence of endometrium-like tissue outside the uterus such as the pelvic peritoneum, ovaries and rectovaginal septum. Three types of endometriosis lesions can be distinguished: superficial (mainly peritoneal) endometriosis, deep endometriosis (nodules) and ovarian endometriotic cysts (endometriomas). Surgical treatment improves the quality of life with reduction of pain and optimalization of spontaneous pregnancy rates. The surgical treatment of endometriomas can be performed by different techniques. Although the surgical treatment of endometriomas is widely accepted, the ideal surgical technique is still being debated.

There are two main risks associated with the surgical treatment of endometriomas:

1. Removal or destruction of normal ovarian cortex together with the endometrioma with subsequent reduction of ovarian reserve.
2. Incomplete surgery with subsequent early recurrence of endometriomas.

With the BLAST study we want to compare two different conservative surgical techniques for the treatment of endometriomas: the combined technique versus CO2 laser vaporization only. Both are existing and accepted surgical strategies in the treatment of endometriomas. For both techniques fenestration and drainage of the cysts is performed.

- The combined technique: first step consisting of stripping the cyst wall for 80% of the surface, followed by a second step consisting of ablation of the remaining 20% cyst surface attached to the ovarian vascular hilus.
- CO2 laser vaporization only: ablation of the entire cyst wall using the CO2 laser.

The ovarian reserve is the stock of follicles in the ovaries of a women at a certain moment in her life. The initial stock is laid out during fetal life (in the womb). This ovarian reserve/stock decreases progressively with the age of a women. The ovarian reserve can be measured using different techniques, mostly hormonal dosage and transvaginal ultrasound. In this study, hormonal dosage of AMH (Anti-Mullerian hormone) was chosen because it is, at this moment, the most reliable marker of the ovarian reserve. The Anti-Mullerian hormone can be measured at each moment in the menstrual cycle and the result is an objective value. Ultrasound evaluation of a cyst is cycle dependent and is limited by the presence of the cyst. Although both surgical techniques used in this study are known to preserve the ovarian tissue as much as possible, the ideal technique concerning ovarian reserve is still unknown.

The effect of both surgical techniques on the ovarian reserve will be measured at different timepoints as reflected by the level of AMH in the blood (AMH is an ovarian reserve marker). Additionally, recurrence rate, pregnancy rates en evolution of pain patterns will be analysed over the time.

The investigators hope that this randomized (this means that fate determines in which treatment group you will be divided) clinical study can eventually offer benefits to patients in the same situation as you are, namely young women diagnoses with endometriosis with possible childwish in the future.

UZ Leuven is the sponsor of this study and the study will be conducted in 4 different hospitals in Belgium (UZ Leuven – Leuven, CHR La Citadelle – Liege, GZA Sint-Augustinus – Antwerp and Cliniques Universitaires Saint-Luc (UCL) - Brussels). A total of 92 patients will be included in this study.

Who is eligible:

- Women aged between 18 and 40 years
- Presence of a unilateral endometrioma of ≥2,5cm and ≤8cm

- Presence of a contralateral cyst of ≤2cm is tolerated
- Complaining of infertility and/or pain
- BMI ≤35
- Use of contraception (Progesteron only or combined) for at least 4 weeks before surgery)

Who is not eligible

- <18 years
- >40 years
- Contra-indication for the use of contraception
- Use of GnRH analogues preoperatively and in the first 3 months postoperatively
- (history) of hysterectomy
- History of cancer
- Pituitary/hypothalamic disorders
- Suspected malignancy
- Contralateral endometrioma of ≥2cm
- AMH <0,7 ng/mL preoperatively
- Pregnancy
- Women who do not give written informed consent to participate

Course of the study

The different time moments of the study are listed below, as well as the study procedures.

Pre-screening:

You are eligible for this study when you consult a gynecologist/fertility specialist from a participating center for complaining about pain/infertility with suspicion of underlying endometriosis. The routine examinations for the mapping of the extend of the endometriosis will be done by the standard of care of each participating hospital. This mapping always consist of a transvaginal ultrasound examination.

Screening:

If the transvaginal ultrasounds detects an endometrioma, you are eligible for the study. The standard of care is surgical treatment with measurement of the AMH prior the surgery. The value of the AMH reflects the ovarian reserve. Only if this value is >0,7 ng/mL the study will be presented. If you want to participate in the study, you will be asked to sign the consent form. All examinations for this moment are standard conducted at your hospital.

Treatment:

Your doctor decides to plan a CO2 laser laparoscopy. In preparation of the surgery we ask you to use hormonal contraceptives (Progesteron only or combined) for at least 4 weeks before the surgery. The specific surgical technique (the combined technique or CO2 laser vaporization only) is decided by a computer system based on chance and your doctor has no influence on this.

The day before the planned surgery a standard blood analysis is performed, at this moment the baseline AMH level will also be measured (=study sample).

Follow-up:

Both groups have the same follow-up:

- 3 months postoperatively

- Standard postoperative visit (=standard of care)
- Registration of postoperative complications
- Anamnesis concerning pain, hormonal therapy, potential fertility treatment and child wish (=standard of care)
- Blood analysis with measurement of AMH level (=standard of care)
- Transvaginal ultrasound to detect recurrence (=standard of care)

- 6 and 12 months postoperatively
  - Anamnesis concerning pain, hormonal therapy, potential fertility treatment and childwish (=standard of care)
  - Blood analysis with measurement of AMH level (=study sample)
  - Transvaginal ultrasound to detect recurrence (=standard of care)
- 24 months postoperatively
  - Anamnesis concerning pain, hormonal therapy, potential fertility treatment and childwish (=standard of care)
  - Transvaginal ultrasound to detect recurrence (=standard of care)

If you are pregnant or if fertility treatment with use of GnRH analogues is used, you drop out of the study. The data that have been collected so far, will be used for the analysis.

Your participation in the study will last around 30 months and involve no extra visits in addition to those involved in your care if you do not take part in the study.

Similarly, several additional examinations or procedures will be required in connection with the study.

Since your participation in the study is part of the care of your clinical situation, some of the visits and examinations we will describe are part of the normal care provided in your hospital, while others are offered by the study.

Overview table of the course of the study:

Eligibility assessment	Visits						
	Screening	Baseline*	Hospitalization	Follow up			
				3 months	6 months	12 months	24 months
History/clinical exam	x						
Informed consent		x					
Eligibility assessment		x					
Randomization		x					
Laparoscopic surgery (group 1 vs group 2)			x				
Postoperative check-up				x			
Complication assessment				x			
Anamnesis				x	x	x	x
Ultrasound				x	x	x	x
AMH-value	x		D-1*	x	x*	x*	

* study related visits/samples

<u>Schematic presentation of the course of the study:</u>

STUDY FLOW CHART

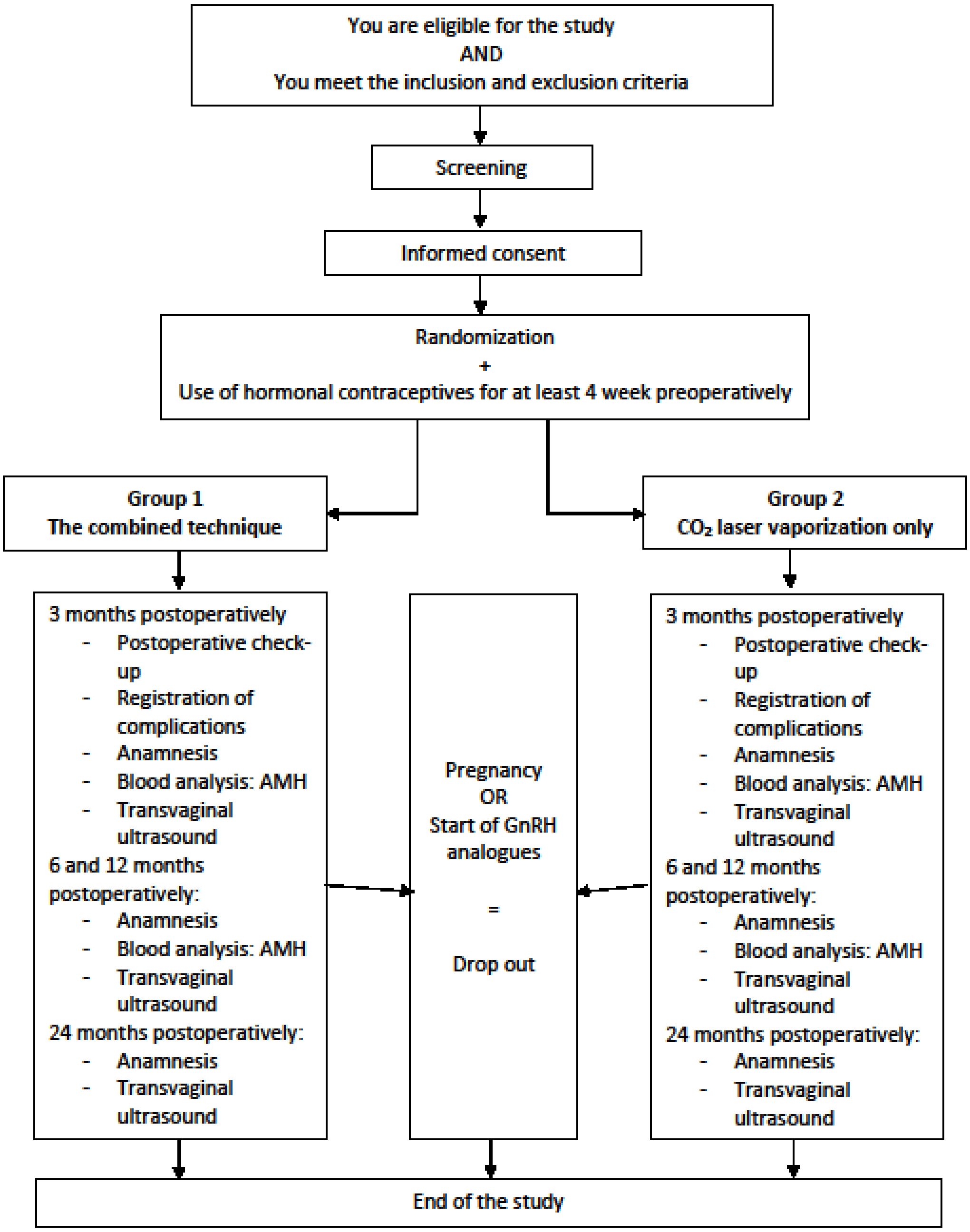

Risks and discomforts

The study proposed to you has no extra risk. Both surgical techniques are existing and accepted surgical strategies.

Risks associated with the surgical procedure (cf. separate informed consent concerning the planned surgery based on the preoperative examinations):

- during the laparoscopic procedure, the fertility surgeon may decide to proceed via open abdominal surgery, which will be performed during the same narcosis.
- there is a slight chance that problems occur after the operation with the urinary tract, bladder, intestine or blood vessels (leakage, fistula, bleeding, infection, thrombosis, …). The involvement of an abdominal surgeon, urologist or vascular surgeon may be required to address these problems properly.

Risks associated with the procedures of the clinical study:

The taking of blood for the analysis of the Anti-Mullerian Hormone (AMH) may (rarely) cause pain, bleeding, bruising or infection localised around the injection site. Similarly, some patients may feel dizzy or even faint during the procedure. The staff who take the blood will do all they can to keep these discomforts to a minimum.

Benefits

If you decide to participate, this has no benefits or disadvantages for you. The information gathered from this research may contribute to the implementation of a scientific based decision making for women in the same situation as you are.

Consent and refusal

Your participation is voluntary, and you are entitled to withdraw from the study for any reason, without having to justify your decision. Nevertheless, it may be useful for the investigator and for the sponsor of the study to know if you are withdrawing because the constraints of the treatment are too great (too many uncomfortable side effects, for example).

It is also possible that the investigator withdraws you from the study because you are pregnant, because he/she thinks it is better for your health or because he/she finds out that you are not following the instructions given to participants.

Finally, the competent national or international authorities, the ethics committee that initially approved the study or the sponsor may break off the study because the information gathered shows that the investigational treatment is not effective (does not deliver a sufficient level of improvement in the health of the participants), the investigational treatment causes more side effects or more serious side effects than anticipated, or for any other reason, such as, for example, the decision to stop research and development of the investigational medicinal product.

Samples of biological material collected during the study

The sponsor of the study undertakes that the samples will only be used within the context defined in the section “Progress of clinical research” and its appendices.

Samples collected for the analyses described for the study in this document. The surplus of your samples will be destroyed once the analyses described in this document have been carried out.

You should also be aware that:

For your safety, it is advisable for your GP, if you have one, or other specialists in charge of your health to be informed of your participation in this study. We will ask you to confirm your agreement, but will respect your wish not to inform them where applicable.

Contact

If you need further information, but also if you have problems or concerns, you can contact the investigator (…………………………………..) or a member of his/her research team (………………………) on the following email address: ……………………………………….. or you can contact the coordinating center: Dr Celine Bafort on the following email address: celine.bafort@uzleuven.be of by telephone number (016340088).

In case of emergency, contact the A&E department of your hospital, indicating that you are taking part in a clinical study. Your records will contain information of use to the on-call doctor in relation to this clinical study.

If you have any questions relating to your rights as a participant in a clinical study, you can contact the patient rights ombudsman of your institution on this telephone number: …………………….. If necessary, he/she can put you in contact with the ethics committee.

Title of the study: BLAST: A randomized controlled trial on conservative endometrioma surgery using the CO2 laser: the combined technique versus CO2 laser vaporization only.

II Informed consent

Participant

I declare that I have been informed of the nature of the study, its purpose, its duration, any risks and benefits and what is expected of me. I have taken note of the information document and the appendices to this document.

I have had sufficient time to think about it and discuss it with a person of my choice, such as my GP or a member of my family.

I have had the opportunity to ask any questions that came to mind and have obtained a satisfactory response to my questions.

I understand that my participation in this study is voluntary and that I am free to end my participation in this study without this affecting my relationship with the therapeutic team in charge of my health.

I understand that data about me will be collected throughout my participation in this study and that the investigator and the sponsor of the study will guarantee the confidentiality of these data in accordance with applicable European and Belgian legislation.

I agree to my personal data being processed as described in the section dealing with confidentiality guarantees (appendix).

I agree/do not agree (delete as appropriate) to the study data collected for the purposes of this study being processed at a later date provided this processing is limited to the context of the present study for a better understanding of the disease and its treatment.

I agree/do not agree (delete as appropriate) to my GP or other specialists in charge of my health being informed of my participation in this clinical study.

I have received a copy of the information to the participant and the informed consent form.

Surname, first name of the volunteer:

Date:

Signature:

Investigator

I, the undersigned, ……………………………………… investigator, confirm that I have verbally provided the necessary information about the study and have given the participant a copy of the information document.

I confirm that no pressure was applied to persuade the patient to agree to take part in the study and that I am willing to answer any additional questions if required.

I confirm that I operate in accordance with the ethical principles set out in the latest version of the "Helsinki Declaration", the "Good Clinical Practices" and the Belgian Law of 7 May 2004 related to experiments on humans.

Name, Surname of the investigator:

Date:

Signature:

Title of the study: BLAST: A randomized controlled trial on conservative endometrioma surgery using the CO2 laser : the combined technique versus CO2 laser vaporization only.

III Supplementary information

Supplementary information on the protection and the rights of the participant in a clinical study

Ethics Committee

This study has been reviewed by an independent Ethics Committee, namely the Ethische commissie onderzoek UZ/KULeuven (EC onderzoek), which has issued a favourable opinion after consulting with the Ethics Committees of each centre in Belgium where this trial will be conducted. It is the task of the Ethics Committees to protect people who take part in a clinical trial. They make sure that your rights as a patient and as a participant in a clinical study are respected, that based on current knowledge, the balance between risks and benefits remains favourable to the participants, that the study is scientifically relevant and ethical.

You should not under any circumstances take the favourable opinion of the Ethics Committee as an incentive to take part in this study.

Voluntary participation

Before signing, do not hesitate to ask any questions you feel are appropriate. Take the time to discuss matters with a trusted person if you so wish.

Your participation in the study is voluntary and must remain free of any coercion: this means that you have the right not to take part in the study or to withdraw without giving a reason, even if you previously agreed to take part. Your decision will not affect your relationship with the investigator or the quality of your future therapeutic care.

However, it is advisable for your safety to inform the investigator if you have decided to stop taking part in the study.

If you agree to take part, you will sign the informed consent form. The investigator will also sign this form to confirm that he/she has provided you with the necessary information about the study. You will receive a copy of the form.

Costs associated with your participation

The costs of the study related blood samples (for measurement of AMH level) will be paid by the investigator/sponsor.

You won’t receive any financial compensation for participation in this study. If you decide to take part in this study, this will not therefore involve any extra costs for you or your insurer. The visits and procedures identified as specific to the study in the description of the course of the study on page 3 to 5 will be paid for by the sponsor. You may only be charged for the costs corresponding to the standard medical care in your clinical situation.

Guarantee of confidentiality

Your participation in the study means that you agree to the investigator collecting data about you and to the study sponsor using these data for research purposes and in connection with scientific and medical publications. [If another legal basis is chosen than consent, use:] Your participation in the study means that your personal data are collected by the investigator and used in an encoded form by the study sponsor for research purposes and in connection with scientific and medical publications.

Your data will be processed in accordance with the European General Data Protection Regulation (GDPR). The sponsor shall act as data controller for your data. You are entitled to ask the investigator what data are being collected about you and what is their use in connection with the study. This data concerns your current clinical situation but also some of your background, the results of examinations carried out within the context of care of your health in accordance with the current standards and

obviously the results of examinations required by the protocol. You have the right to inspect these data and correct them if they are incorrect[1].

The investigator has a duty of confidentiality vis-à-vis the data collected.

This means that he/she undertakes not only never to reveal your name in the context of a publication or conference but also that he/she will encode (your identity will be replaced by an ID code in the study) your data before sending them to the manager of the database of collected data.

The investigator and his/her team will therefore be the only ones to be able to establish a link between the data transmitted throughout the study and your medical records[2].

The personal data transmitted will not contain any combination of elements that might allow you to be identified[3].

For the study data manager designated by the sponsor, the data transmitted will not allow you to be identified. The latter is responsible for collecting the data gathered by all investigators taking part in the study, processing them and protecting them in accordance with the requirements of the Belgian law on the protection of privacy.

To verify the quality of the study, it is possible that your medical records will be examined by persons subject to professional secrecy and designated by the ethics committee, the sponsor of the study or an independent audit body. In any event, this examination of your medical records may only take place under the responsibility of the investigator and under the supervision of one of the collaborators designated by him/her.

The (encoded) study data will be able to be sent to the relevant ethics committees or to other doctors working in collaboration with the sponsor.

They will also be able to be sent to other sites of the sponsor in Belgium. As explained above, the transmitted data are encoded[4].

Your consent to take part in this study therefore also implies the use of your encoded medical data for the purposes described in this information form and to their transmission to the aforementioned people and authorities.

The sponsor undertakes only to use the data collected within the context of the study in which you are taking part.

If you withdraw your consent to take part in the study, to guarantee the validity of the research, the data encoded up to the point at which you withdraw will be retained. No new data may be sent to the sponsor.
If you have any questions relating to how your data are being processed, you may contact the investigator. The data protection officer in your hospital can be contacted as well: DPO - UZ Leuven, Herestraat 49, 3000 Leuven, e-mail dpo@uzleuven.be.

Finally, if you have a complaint concerning the processing of your data, you can contact the Belgian supervisory authority who ensures that privacy is respected when personal data are processed.

The Belgian supervisory authority is called:
Data Protection Authority (DPA)
Drukpersstraat 35,
1000 Brussels
Tel. +32 2 274 48 00
e-mail: contact@apd-gba.be
Website: https://www.dataprotectionauthority.be

---

[1] These rights are guaranteed by the European Data Protection Regulation (GDPR) and by the Law of 22 August 2002 on patient rights.

[2] For clinical trials, the law requires this link with your records to be retained for 20 years. In the case of a advanced therapy medicinal product using human biological material, this period will be a minimum of 30 years and a maximum of 50 years in accordance with the Belgian Law of 19 December 2008 on the use of human biological material and the applicable royal decrees.

[3] The database containing the results of the study will therefore not contain any combination of elements such as your initials, your gender and your full date of birth (dd/mm/yyyy).

[4] The sponsor then undertakes to respect the constraints of the European General Data Protection Regulation (GDPR) and the Belgian legislation on the protection of natural persons with regard to the processing of personal data.

Future of your sample(s) collected during the study

The sample encoding procedure is the same as that used for your medical data. Samples sent to the sponsor will therefore only include your study ID code.

The manager of these samples (laboratoriumgeneeskunde UZ Leuven) undertakes to use them within the context of clinical research and to destroy them at the end of the scheduled storage period.

The sample of biological material taken is deemed to be a "donation" and you should be aware that, in principle, you will not receive any financial benefit (royalties) associated with the development of new therapies derived from the use of your donation of biological material and which may be of commercial value.

If you withdraw your consent to take part in the study, you may contact the investigator and have those of your samples that have not yet been used destroyed. The results obtained from your samples before you withdraw your consent remain the property of the study sponsor.

Insurance

Any participation in a clinical study involves a risk, however small it is. Even if there is no fault, the sponsor accepts responsibility for damage caused to the participant (or in the event of death, his/her dependants) and directly or indirectly linked to his/her participation in the study. The sponsor has taken out insurance for this responsibility[5].

If the investigator believes that a link with the study is possible (the insurance does not cover the natural progression of your disease or the known side effects of your normal treatment), he/she will inform the study sponsor, which will initiate the declaration procedure to the insurance company. The latter will appoint an expert - if it considers it necessary - to assess whether there is a link between your new health problems and the study.

In the event of disagreement either with the investigator or with the expert appointed by the insurance company and also whenever you feel it is appropriate, you or - in case of death - your dependants may bring proceedings against the insurer directly in Belgium (Amlin Insurance SE, met adres Plantin en Moretuslei 297 te Antwerpen, onder polis nummer 299.053.700.). The law provides that the insurer may be summoned to appear either before the judge of the location where the event giving rise to the damage occurred, or before the judge of your domicile, or before the judge of the insurer's registered offices.

[5] In accordance with Article 29 of the Belgian Law related to experiments on humans (7 May 2004)
